# *SMARCA4*-inactivating mutations increase sensitivity to Aurora kinase A inhibitor VX-680 in non-small cell lung cancers

**DOI:** 10.1038/ncomms14098

**Published:** 2017-01-19

**Authors:** Vural Tagal, Shuguang Wei, Wei Zhang, Rolf A. Brekken, Bruce A. Posner, Michael Peyton, Luc Girard, TaeHyun Hwang, David A. Wheeler, John D. Minna, Michael A. White, Adi F. Gazdar, Michael G. Roth

**Affiliations:** 1 Department of Biochemistry, UT Southwestern, Dallas, Texas 75390, USA; 2 Department of Pathology, UT Southwestern, Dallas, Texas 75390, USA; 3 Hamon Center for Therapeutic Oncology Research, UT Southwestern, Dallas, Texas 75390, USA; 4 Department of Pharmacology, UT Southwestern, Dallas, Texas 75390, USA; 5 Harold Simmons Comprehensive Cancer Center, UT Southwestern, Dallas, Texas 75390, USA; 6 Department of Clinical Sciences, UT Southwestern, Dallas, Texas 75390, USA; 7 Department of Molecular and Human Genetics, Baylor College of Medicine, Houston, Texas 77030, USA; 8 Department of Medicine, UT Southwestern, Dallas, Texas 75390, USA; 9 Department of Cell Biology, UT Southwestern, Dallas, Texas 75390, USA

## Abstract

Mutations in the *SMARCA4/BRG1* gene resulting in complete loss of its protein (BRG1) occur frequently in non-small cell lung cancer (NSCLC) cells. Currently, no single therapeutic agent has been identified as synthetically lethal with SMARCA4/BRG1 loss. We identify AURKA activity as essential in NSCLC cells lacking SMARCA4/BRG1. In these cells, RNAi-mediated depletion or chemical inhibition of AURKA induces apoptosis and cell death *in vitro* and in xenograft mouse models. Disc large homologue-associated protein 5 (HURP/DLGAP5), required for AURKA-dependent, centrosome-independent mitotic spindle assembly is essential for the survival and proliferation of *SMARCA4/BRG1* mutant but not of * SMARCA4/BRG1* wild-type cells. AURKA inhibitors may provide a therapeutic strategy for biomarker-driven clinical studies to treat the NSCLCs harbouring *SMARCA4/BRG1*-inactivating mutations.

## Abstract (web summary)

Lung cancers often harbour loss-of-function mutations in *SMARCA4*. Here, the authors demonstrate a vulnerability of *SMARCA4*-deficient lung cancers for Aurora kinase A inhibition associated with mitotic defects.

The goal of personalized cancer medicine is to effectively match the correct therapy with the correct patient. However, success in this approach has been limited owing to lack of adequate prognostic methods^1^,^2^. Lung cancer is a prime example of marginal success in cancer therapeutics. Despite the discovery of 14 novel anticancer agents and a total of 20 Food and Drug Administration-approved drugs in use, the mortality associated with this disease has not changed significantly in the past quarter century^1^. This is in part due to the fact that until recently therapeutic strategies in patient care have considered large patient populations as a single group owing to lack of understanding of interindividual variations relevant to the disease. It is becoming clear that the ability to identify susceptible patient subtypes for each of the existing therapeutic strategies is as essential as the discovery of new-generation anticancer therapeutic agents. Pharmacogenomics provides the potential to fulfill this need by identifying genetic changes in tumours that can be linked to ‘druggable' vulnerabilities. Therefore, current research in the practice of oncology attempts to build a reliable database of linkages between genetic repertoires of tumours and existing therapeutic strategies^3^.

The first successes of genomics-driven cancer therapeutics targeted mutant oncoproteins driving tumour growth^4^. Two examples are therapies inhibiting epidermal growth factor receptor (EGFR) in lung tumours bearing EGFR-activating mutations^5^,^6^ and ALK in tumours driven by ALK fusion proteins^7^. In addition to targeting mutations that drive tumour growth, it is theoretically possible to identify acquired vulnerabilities in cells linked to the loss of tumour suppressors such as *SMARCA4*, one of the more commonly mutated tumour suppressors in lung cancers.

Somatic mutations in *SMARCA4*, the gene encoding BRG1 (henceforth SMARCA4), occur in 31 of the 159 lung cancer-derived cell lines (19%) in the COSMIC database or sequenced by us (authors' unpublished data). Other studies have reported the frequency of SMARCA4-inactivating mutations in non-small cell lung cancer (NSCLC) cell lines ranging from 15% to 35% (refs 8, 9), 5–10% in lung adenocarcinomas^10^,^11^,^12^ and 6% in lung squamous cell carcinomas^13^. Mutations in SMARCA4 in tumours are reported less frequently than in cell lines. However, current sequencing technology often fails to detect large genomic deletions^14^, which frequently occur in SMARCA4-inactivated cancers, thus SMARCA4 inactivation is anticipated to be more common in NSCLC tumours than currently reported. In addition to NSCLCs, accumulating evidence suggests that SMARCA4 also has a tumour-suppressor function for many other cancer types^15^,^16^. Its inactivation is predicted to interfere with the cellular functions of the ATP-dependent SWI/SNF chromatin remodeling complex, as SMARCA4 is one of the two mutually exclusive and non-redundant ATPase subunits of the complex^16^. SWI/SNF complexes mobilize histone octamers in an ATP-dependent manner to allow or repress gene transcription^17^,^18^. Loss of SMARCA4 expression changes approximately 5% of the mammalian transcriptome^19^, including changes in the expression of genes associated with NSCLC^12^, increasing the likelihood for loss of functions that might be redundant in normal cells and non-redundant in tumours lacking SMARCA4.

To identify targetable gene products related to *SMARCA4*-inactivating mutations, we applied a high-throughput, cell-based, one-well/one-gene screening platform with a genome-wide library of chemically synthesized small interfering RNAs (siRNAs) looking for essential genes in SMARCA4-null NSCLC cells that encoded cellular machinery linked to a known drug target. Through this approach, we found that NSCLC cell lines deficient in SMARCA4 are hypersensitive to inhibitors of Aurora kinase A (AURKA), a kinase required for mitotic spindle assembly^20^.

## Results

### Genes essential for survival of SMARCA4-inactivated NSCLCs

**Figure 1:**
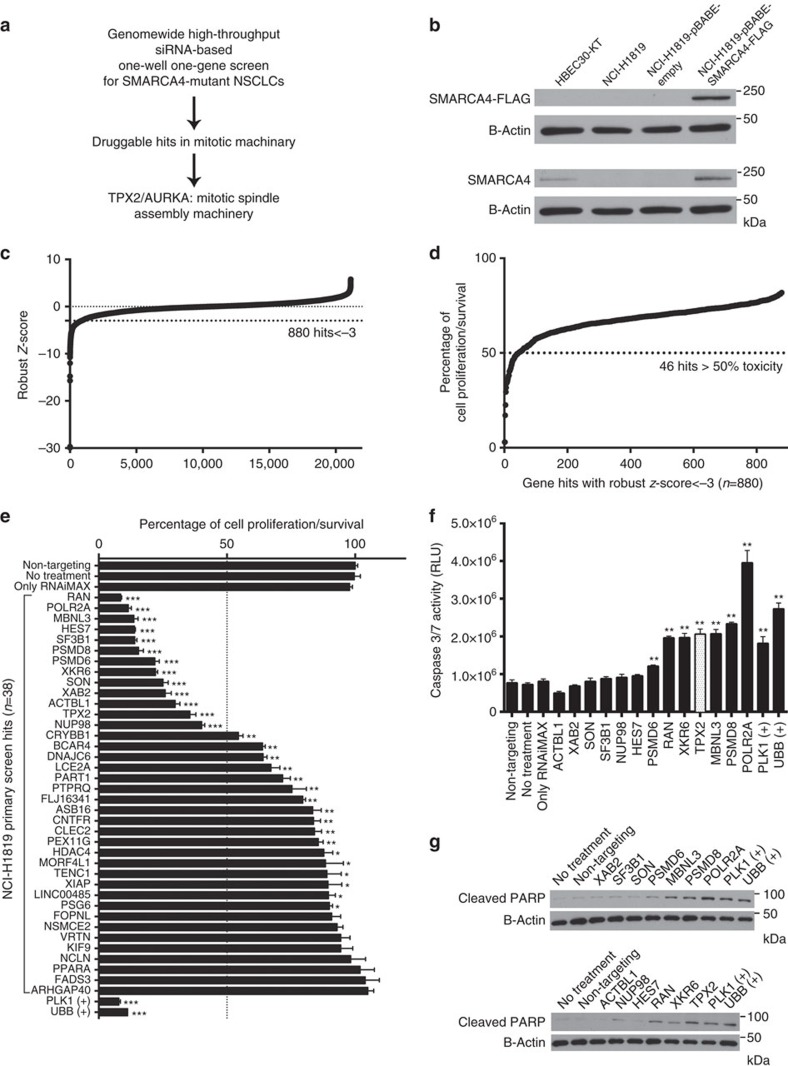
Genome-wide high-throughput siRNA-based screening in NCI-H1819 cells. (**a**) Flow chart of the project. (**b**) NCI-H1819 cells do not express SMARCA4. The endogenous levels of SMARCA4 and ectopic expression of SMARCA4-FLAG in HBEC30-KT, NCI-H1819 and NCI-H1819 infected with pBABE or pBABE-SMARCA4-FLAG were measured by western blotting. (**c**) Schematic representation of the results from screening pooled siRNAs for each of 21,124 human genes in NCI-H1819 cells. Cell viability after the knockdowns was determined with a CellTiter-Glo assay, which measures cellular ATP as an indicator of living cells. Robust *z*-scores for triplicate samples of each siRNA pool were calculated and genes with a *z*-score <−3 were selected as statistically significant. (**d**) Mean viability for triplicate biological replicates of each siRNA pool with a *z*-score <−3 was determined and genes with viability <50% were selected for further analysis. (**e**) Thirty-eight differentially toxic hits were re-analysed and 30 significantly reduced the viability of NCI-H1819 cells (*P*<0.01) compared with non-targeting siRNAs. Error bars on graphs are s.d. of means from triplicate biological replicates. *Indicates statistical significance (*P*<0.01), ** indicates statistical significance (*P*<0.0001) and *** indicates statistical significance (*P*<0.0001) combined with higher biological significance (viability <50%). (**f**) Thirteen siRNA pools showing >50% toxicity and two controls (PLK1 and UBB) were tested in triplicate for activation of caspases 3 and/or 7 with a Caspase-Glo 3/7 assay in NCI-H1819 cells. ** indicates siRNAs significantly (*P*<0.01) activating caspases compared with non-targeting siRNAs. Among these, TPX2 and RAN are functionally related to AURKA and TPX2 (white bar) is a direct binding partner and activator of AURKA. Error bars indicate s.d. of triplicate biological replicates. (**g**) Western blot analysis of these 13 siRNA pools with monoclonal antibodies for cleaved PARP and β-Actin confirmed the apoptotic response in 7 of them by an orthogonal assay. PLK1 (+) and UBB (+) indicate positive controls that are universally toxic siRNAs. Statistical significance in the figures was assessed by one-way ANOVA and *post hoc* Dunnett's multiple comparison tests.

To seek candidates for targeted therapy that are synthetically lethal to loss of *SMARCA4* in NSCLC cells, we conducted a whole-genome siRNA library screen in a cell line belonging to a panel of NSCLC-derived cell lines that has been extensively characterized^21^. From the cell lines harbouring homozygous *SMARCA4*-inactivating mutations, we chose NCI-H1819 because it had no detectable wild-type SMARCA4 protein (Fig. 1b) and lacked mutations in other oncogenic drivers or tumour suppressors, including *KRAS*, *EGFR*, *PIK3CA*, *BRAF*, *PTEN*, *RB1*, *STK11*, *TP53* and *CDKN2A*^22^, most commonly detected as mutant in NSCLCs, and might therefore produce a phenotype more directly reliant on loss of SMARCA4.

siRNA transfections were performed in triplicate with pools of 50 nM of four separate siRNA duplexes targeting each of 21,124 genes and cell viability was measured after 96 h. We identified 880 siRNA pools with *z*-scores <−3 (Fig. 1c, Supplementary Data 1), from which we focussed on the top 46 genes whose depletion inhibited the growth or survival of NCI-H1819 cells by >50% (Fig. 1d). From this list, we excluded eight genes that had been found to be toxic in a previous screen performed with a wild-type *SMARCA4*-expressing immortalized human bronchial epithelial cell (HBEC) line, HBEC30-KT (Supplementary Data 1 and 2)^23^. We performed a secondary validation assay with fresh pools of siRNAs targeting the remaining 38 genes and 30 of the 38 reiteratively confirmed the toxic phenotype (Fig. 1e). Thirteen of these 30 validated siRNA pools inhibited cell proliferation or survival by >50% and were studied in detail.

**Figure 2:**
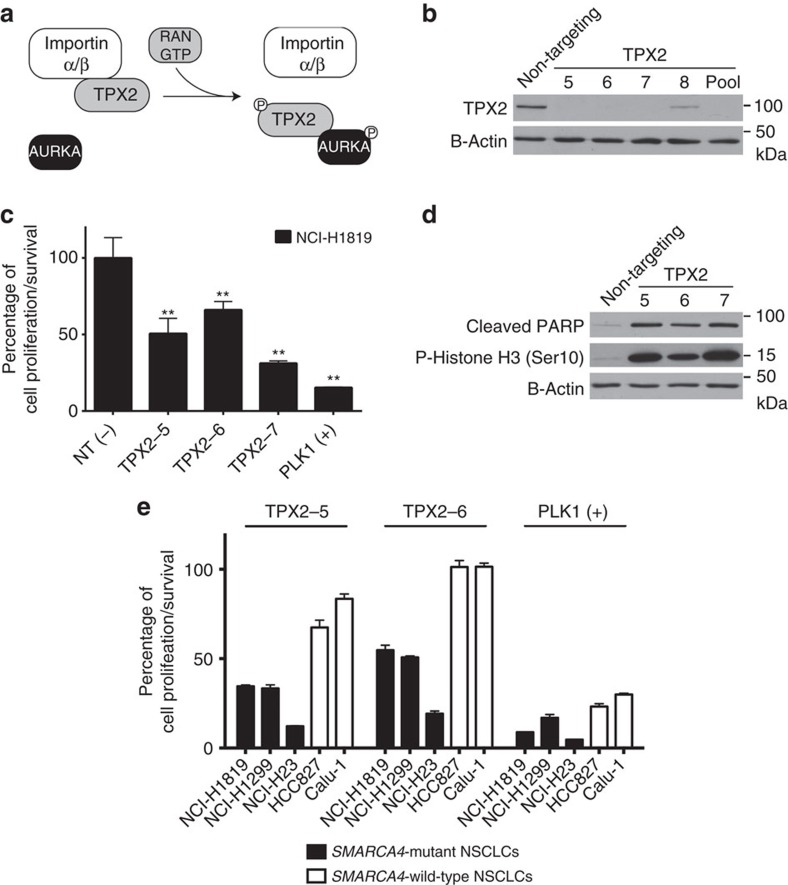
Loss of TPX2 induces both apoptosis and mitotic arrest in NCI-H1819 cells. (**a**) Cartoon showing that TPX2 and RAN act sequentially to activate AURKA, a known regulator of mitosis. RAN prevents the inhibitory interaction of Importins on TPX2, allowing it to activate AURKA for mitotic spindle assembly. (**b**) Immunoblot analysis confirms that three of the four siRNAs greatly reduce TPX2 protein levels in NCI-H1819 cell lysates 3 days after transfecting the cells. (**c**) Five days after transfecting NCI-H1819 cells with non-targeting or individual siRNAs targeting TPX2, cell viability was measured with a CellTiter-Glo viability assay. PLK1 was depleted as the positive control. NT=nontargeting. ** indicates statistical significance (*P*<0.001). Statistical significance was assessed by one-way ANOVA and *post hoc* Dunnett's multiple comparison tests. (**d**) The effect of individual siRNAs on NCI-H1819 cells was measured by immunoblotting with monoclonal antibodies to cleaved poly (ADP-ribose) polymerase 1 (PARP), phospho-Histone H3 and β-Actin as a loading control 3 days after transfecting the cells with either non-targeting or TPX2-targeting siRNAs. Cleaved PARP indicated an active apoptotic response and phospho-Histone H3 indicated mitotic arrest. Data are representative of duplicate experiments. (**e**) Five days after transfecting NCI-H1819, NCI-1299, NCI-H23, HCC827 and Calu-1 cells with non-targeting or individual siRNAs #5 and #6 targeting TPX2, cell viability was measured with a CellTiter-Glo assay that measure cellular ATP as a surrogate for cell proliferation or survival. PLK1 was depleted as the positive control. Error bars on graphs are s.d. of means from triplicate biological replicates.

To distinguish cytotoxic from cytostatic effects, we examined whether these genes, when depleted, activated caspases 3 and/or 7 and induced cleavage of poly(ADP-ribose) polymerase 1 (PARP1) as surrogate markers of apoptosis (Fig. 1f,g). Seven of these 13 siRNA pools induced apoptosis in NCI-H1819 cells. In this experiment, siRNAs for the Polo-like kinase 1 (PLK1) and Ubiquitin B were included as positive controls as we have commonly detected them as toxic siRNAs in screens of NSCLC cell lines. Previous reports showed that inactivation of SMARCA4 in cells derived from non-cancerous tissue causes mitotic catastrophe as the primary defect^24^. Among the seven cytotoxic siRNA pools for NCI-H1819 cells, TPX2 and RAN were of immediate interest because they function together in mitosis to regulate AURKA (Fig. 2a), for which specific chemical inhibitors have been described^25^,^26^.

### TPX2 is required by NSCLC cells with inactivated *SMARCA4*

To exclude the possibility of off-target effects with the pooled siRNA duplexes in the library, we obtained individual siRNAs to confirm our screening results with TPX2 knockdown. We first checked TPX2 protein levels after transfection with these individual siRNAs (Fig. 2b). Three of the four siRNAs successfully reduced the TPX2 protein (Fig. 2b) and those siRNAs individually induced significant toxic effects (Fig. 2c) and induced PARP cleavage (Fig. 2d). To investigate the effect of depleting TPX2 on mitosis, we determined the extent of phosphorylation of Histone H3, which is phosphorylated only during mitosis^27^. In the unsynchronized cell population treated with a non-targeting siRNA, very low levels of phosphorylated Histone H3 were observed, consistent with 2–3% of the cell population undergoing mitosis at any time. However, cell samples treated with each of the three siRNAs targeting *TPX2* showed a large increase in Histone H3 phosphorylation (Fig. 2d). This suggested that lack of TPX2 resulted in delayed exit from or cell cycle arrest in mitosis. To expand our observations to a larger panel of NSCLC lines, we tested two of the most efficacious individual siRNAs targeting *TPX2* on an additional two *SMARCA4*-mutant and two *SMARCA4*-wild-type NSCLC lines. As the positive control, in this and following cell viability experiments for gene knockdowns, the depletion of the mitotic kinase PLK1 was used to monitor a general sensitivity to inhibitors of mitosis. Our results showed that both siRNAs targeting *TPX2* were more toxic in *SMARCA4*-mutant lines in comparison to the NSCLC lines harbouring wild-type *SMARCA4* (Fig. 2e). The cells expressing wild-type *SMARCA4* were not simply less sensitive to inhibitors of mitosis, as all of these NSCLC cell lines were similarly sensitive to the depletion of *PLK1*. We further measured the cell doubling times of 26 NSCLC and HBEC lines used in this study, but we did not observe a statistically significant difference (*P*=0.08, one-way analysis of variance (ANOVA) and *post hoc* Dunnett's multiple comparison tests) in the average doubling times between SMARCA4-null and SMARCA4-wild-type NSCLC lines (Supplementary Table 1).

### SMARCA4 loss sensitizes to depletion or inhibition of AURKA

**Figure 3:**
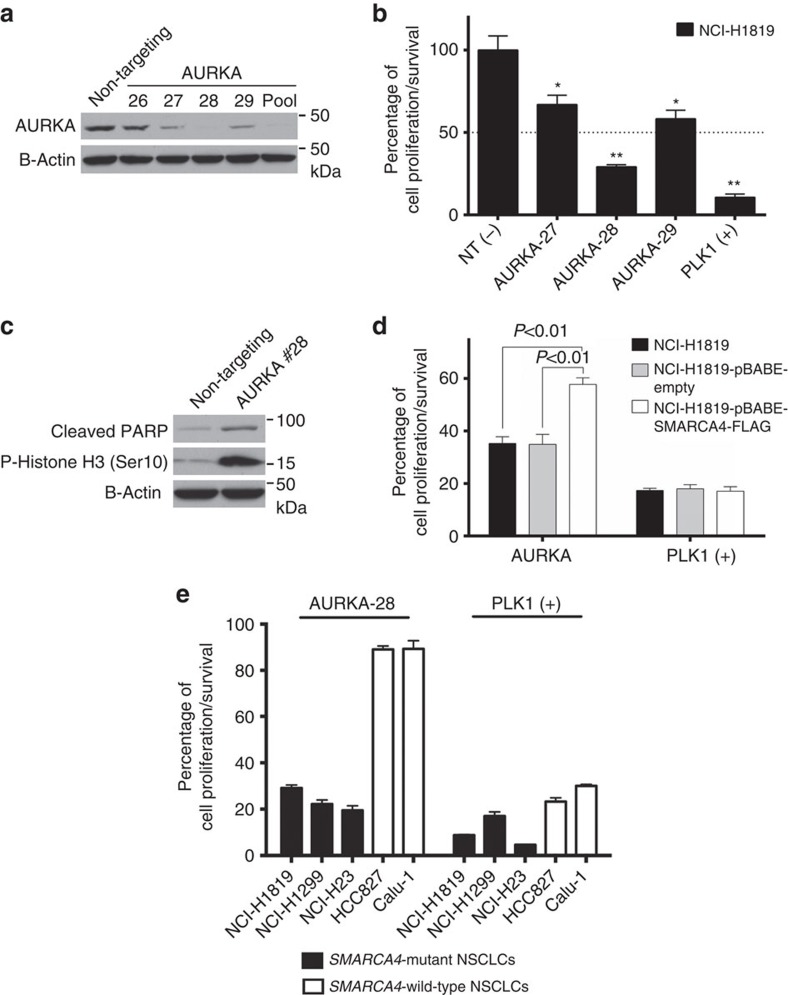
Loss of SMARCA4 expression in NCI-H1819 increases toxicity of siRNAs to AURKA. (**a**) NCI-H1819 cell lysates were collected 3 days after transfecting the cells with either non-targeting or AURKA-targeting siRNAs and were immunoblotted to monitor AURKA protein levels. (**b**) Five days after transfecting NCI-H1819 cells with individual siRNAs, cell viability was measured with a CellTiter-Glo assay on triplicate biological replicates. PLK1 knockdowns were used as the positive control. Means with s.d. are shown on the graphs. * indicates statistical significance (*P*<0.001) and ** indicates statistical significance (*P*<0.001) combined with higher biological significance (viability<50%). (**c**) The response to depletion of AURKA was assessed by immunoblotting with monoclonal antibodies to cleaved PARP, phospho-Histone H3 and β-Actin. (**d**) Five days after depleting AURKA in NCI-H1819, NCI-H1819-pBABE and NCI-H1819-pBABE-SMARCA4-FLAG cells, cell viability was measured with a CellTiter-Glo assay as in part (**b**). (**e**) Five days after transfecting NCI-H1819, NCI-1299, NCI-H23, HCC827 and Calu-1 cells with non-targeting or individual siRNA #28 targeting AURKA, cell viability was measured with a CellTiter-Glo viability assay as in Fig. 2. PLK1 was depleted as the positive control. Error bars on graphs are s.d. of means from triplicate biological replicates. Statistical significance on graphs was assessed by one-way ANOVA and *post hoc* Dunnett's multiple comparison tests.

As TPX2 binds and activates AURKA in mitosis, we depleted AURKA protein with four individual siRNAs to identify the most efficient ones for further experiments (Fig. 3a). Among four siRNAs, only one showed complete knockdown of AURKA, whereas two of the four resulted in partial depletion. Only the most efficient siRNA produced >50% reduction in cell growth, indicating that low levels of AURKA support cell viability (Fig. 3b). Because of its higher efficacy, we used siRNA #28 to deplete AURKA in the following experiments. Depleting AURKA in NCI-H1819 cells induced mitotic arrest and apoptosis (Fig. 3c). To understand whether sensitivity to AURKA depletion is causatively linked with SMARCA4 loss, we restored wild-type *SMARCA4* expression in the NCI-H1819 cell line (Fig. 1b) and performed identical cell toxicity assays with both parental and SMARCA4-expressing NCI-H1819 cells. Expression of exogenous SMARCA4 significantly reduced the response to AURKA knockdown (Fig. 3d) and VX-680 (Supplementary Fig. 10). We also determined whether the growth rates of NCI-H1819 cells changed after introducing wild-type *SMARCA4*. NCI-H1819 cells transduced with the retroviral vector expressing wild-type SMARCA4 had a doubling time of 56.9 h, H1819 cell lines transduced with the empty retroviral vector 56.1 h and parental NCI-H1819 cells 54.5 h. Thus the reduced sensitivity to AURKA depletion in H1819-expressing SMARCA4 was not due to a decrease in the frequency of entering mitosis.

Next we depleted *AURKA* in four additional NSCLC lines, two *SMARCA4* mutant and two *SMARCA4* wild-type, which were previously tested with siRNAs against *TPX2*. We observed that *SMARCA4* mutant lines had significant decrease in cell growth or survival when AURKA was depleted, whereas SMARCA4 wild-type lines were insensitive (Fig. 3e). SMARCA4 status did not determine sensitivity to the other toxic siRNA pool targeting mitotic kinase *PLK1* and thus did not cause a general sensitivity to inhibition of mitosis. These results suggested that SMARCA4 loss might specifically sensitize cells to AURKA-targeted therapies.

To examine this possibility, NCI-H1819 cells were treated with five structurally distinct, commercially available AURKA inhibitors, all of which have been in clinical trials. NCI-H1819 cells were sensitive to all of these, with VX-680 and MLN8237 being the most potent at low nanomolar concentrations (Table 1). However, well above the EC50, at high nanomolar and low micromolar concentrations, MLN8237 showed an unexpected shift to a cytostatic response from cytotoxic, suggesting that it might have additional targets that might be more effective in inducing cell cycle arrest than cell death (Supplementary Fig. 1a). Considering the validated advantages of sustained cytotoxic phenotype over cytostasis in cancer therapies^28^, we chose VX-680 for the following experiments.

**Figure 4:**
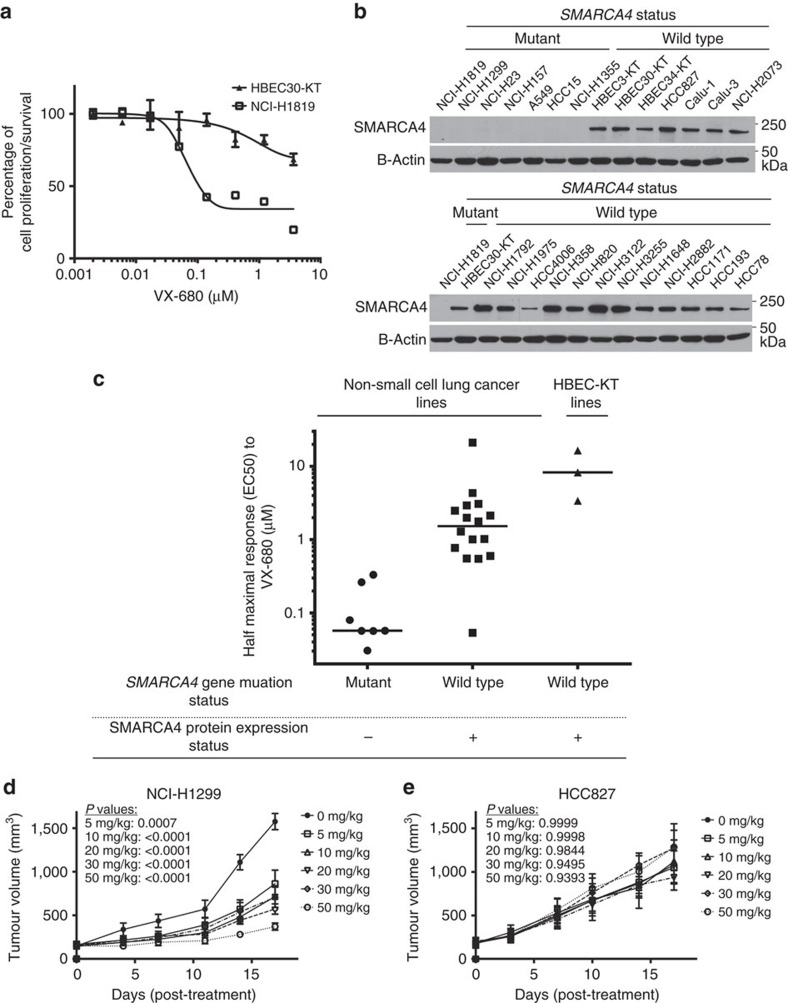
Preferential cytotoxicity of *SMARCA4*-mutant cells after treatment with AURKA inhibitor VX-680. (**a**) NCI-H1819 and immortalized bronchial epithelial cell line HBEC30-KT were treated with serial concentrations of VX-680 and cell viability was measured with a CellTiter-Glo assay. Data are means of triplicate biological replicates with s.d. Some error bars are smaller than the data symbols. (**b**) Cell lysates of 26 NSCLC and HBEC lines were collected and immunoblotted to monitor SMARCA4 levels. SMARCA4-null NCI-H1819 and SMARCA4-wild-type HBEC30-KT were used in both sets of immunoblots as controls. (**c**) A panel of NSCLC and HBEC lines was treated with VX-680 in duplicate, independent dose–response experiments and the mean EC50s of their individual responses are shown. Horizontal bars indicate medians of sample groups. (**d**) SMARCA4-null NCI-H1299 cells and (**e**) SMARCA4-wild-type HCC827 cells were xenografted on NOD/SCID mice. VX-680 treatments were performed i.p. twice daily. For each dosage group (*n*=4), the tumour volume mean and s.e.m. are represented by horizontal line. The significance of the difference between the mean of 0 mg kg^−1^ and of each dose regimen on day 17 was calculated by one-way ANOVA and *post hoc* Dunnett's multiple comparison tests.

VX-680 caused significant toxicity in NCI-H1819 cells with an EC50 of approximately 50 nM (Table 1, Fig. 4a), whereas immortalized normal human bronchial epithelial HBEC30-KT cells were resistant to VX-680 treatment (Fig. 4a). To determine the degree to which SMARCA4 loss correlated with sensitivity to inhibition of AURKA, we measured the sensitivity to VX-680 across a panel of NSCLC and HBEC lines known to be either *SMARCA4*-wild-type or mutant (Supplementary Data 3) and therefore express or lack the SMARCA4 protein, respectively (Fig. 4b). All mutant lines were sensitive to VX-680 compared with NSCLC lines and HBECs expressing wild-type SMARCA4 (Fig. 4c). To determine whether the cytotoxic response to VX-680 in SMARCA4-inactivated NSCLC lines was due to being generally vulnerable to any toxin, in parallel we screened our panel of NSCLC and HBEC lines with seven other drugs belonging to different classes of anticancer agents, as well as with a general chemical toxin (Supplementary Fig. 2a–h, Supplementary Data 3). In this comparative screen, we used the drugs Paclitaxel and GSK923295 as antimitotics inhibiting targets regulating cell division different from VX-680; Pemetrexed and Etoposide as alternative chemotherapy agents targeting nucleotide metabolism and topoisomerase II activity, respectively; Erlotinib and Crizotinib as receptor tyrosine kinase-targeted therapy agents and Bortezomib as an anticancer agent inhibiting proteasome activity. Brefeldin A inhibits vesicle transport from the Golgi to the endoplasmic reticulum, which is vital for all cell types. All of these agents, except Brefeldin A and GSK923295, are Food and Drug Administration-approved for cancer therapies and GSK923295 is a targeted therapy drug candidate currently tested in clinical trials. Dose responses to these nine agents were performed under identical conditions and with identical cell numbers on the same microplate to reduce experimental variables and the experiment was repeated twice on different days. Cell viability was measured after 96 h, and individual EC50 values for each drug/chemical and each cell line were calculated. Mean EC50 values of all cell lines were grouped in SMARCA4 mutant, SMARCA4 wild type and HBEC categories and graphed in Fig. 4c and Supplementary Fig. 2a–h. No correlation between SMARCA4 status of tested cell lines and their response pattern to agents other than VX-680 was observed, indicating that SMARCA4-inactivated NSCLCs are selectively more sensitive to VX-680 but not to other anticancer agents or cellular toxins.

To determine whether this differential toxicity was influenced by conditions of cell culture, we chose two NSCLC lines, one SMARCA4 null, NCI-H1299, and one SMARCA4 wild-type, HCC827, to determine sensitivity to VX-680 in xenografts. These cell lines were selected because of their documented ability to form tumours when xenografted in NOD/SCID mice^29^,^30^. We tested five different dosing regimes in the range of 5 and 50 mg kg^−1^, administered twice daily, to determine the extent of the response to VX-680. NCI-H1299 was highly sensitive to VX-680 treatments, whereas HCC827 was not responsive at any dose (Fig. 4d,e). NCI-H1299 tumours demonstrated significant sensitivity even at a dose of 5 mg kg^−1^, which is lower than previous reports with this agent^25^,^31^,^32^ (Fig. 4d).

### SMARCA4-inactivated NSCLCs require HURP/DLGAP5

**Figure 5:**
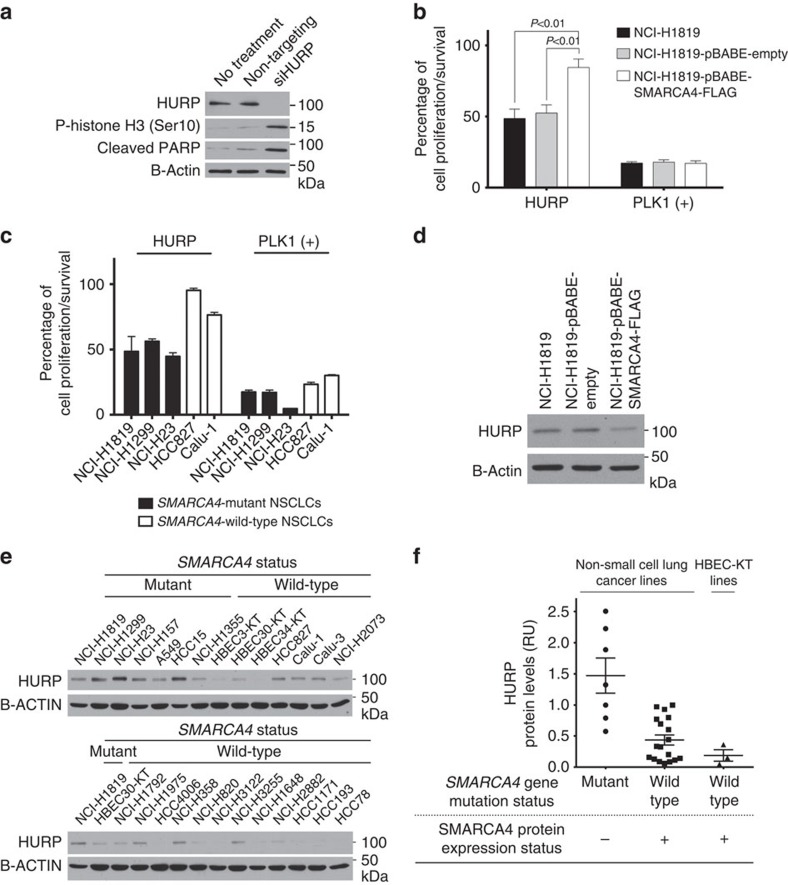
Restoring wild-type SMARCA4 in NCI-H1819 cells reduces the cytotoxicity induced by the depletion of HURP. (**a**) The response to depleting HURP with a pool of four individual siRNAs was measured with monoclonal antibodies to HURP, cleaved PARP (indicating apoptosis) and phospho-Histone H3 (indicating cells engaged in mitosis). (**b**) Five days after depleting HURP in NCI-H1819, NCI-H1819-pBABE and NCI-H1819-pBABE-SMARCA4-FLAG cells and (**c**) in NCI-H1819, NCI-1299, NCI-H23, HCC827 and Calu-1 cells, cell viability was measured with a CellTiter-Glo assay as in Figs 2 and 3. PLK1 was depleted as the positive control. Data are means with s.d. of triplicate biological replicates and representative of triplicate experiments. (**d**) Cell lysates of NCI-H1819, NCI-H1819-pBABE and NCI-H1819-pBABE-SMARCA4-FLAG cells were immunoblotted with monoclonal antibodies to HURP. (**e**) HURP expression was measured in a panel of *SMARCA4*-mutant or wild-type NSCLCs and HBECs by immunoblotting. (**f**) HURP protein band intensities were measured with the ImageQuant software and graphed. Horizontal bars indicate means and s.e.m. of sample groups. Statistical significance on graphs was assessed by one-way ANOVA and *post hoc* Dunnet's multiple comparison tests.

We next sought to understand the basis for the synthetic lethality resulting from AURKA inhibition in SMARCA4-inactivated cells. It has been previously shown that mitotic spindles can be formed by two different mechanisms^20^. Although centrosomes are the organelles responsible for proper mitotic spindle assembly in mammalian somatic cells, a centrosome-independent pathway also exists. Between these two distinct pathways, most of the components overlap. However, the centrosome-independent machinery requires the microtubule-bundling protein, HURP/DLGAP5 (henceforth HURP), while the centrosome-dependent mechanism does not^33^,^34^,^35^. First, we confirmed the efficacy of HURP-targeting siRNAs with immunoblotting and whether knocking down HURP caused cell cycle arrest and apoptosis. HURP depletion in parental NCI-H1819 cells increased phosphorylated Histone H3 and cleaved PARP, indicating cell cycle arrest and induction of apoptosis (Fig. 5a). Consequently, depletion of HURP expression with specific siRNAs resulted in decreased viability in NCI-H1819 cells. However, NCI-H1819 cells with restored wild-type *SMARCA4* were affected significantly less when HURP was depleted (Fig. 5b). We knocked down *HURP* in four additional NSCLC lines, two *SMARCA4* mutant and two *SMARCA4* wild-type, which were previously tested with siRNAs against *TPX2* and *AURKA*. *SMARCA4* mutant lines had significant decrease in cell growth or survival when HURP was depleted, whereas *SMARCA4* wild-type lines were less sensitive (Fig. 5c). This suggests that mammalian cells with SMARCA4 loss may have defects in the centrosome-dependent mitotic spindle mechanism but tolerate this aberration if the centrosome-independent machinery is functional. To understand how HURP regulation changed in the presence of wild-type *SMARCA4* expression, we investigated HURP protein levels in NCI-H1819 cells and SMARCA4-restored NCI-H1819 cells. In comparison to parental or pBABE-Empty vector expressing NCI-H1819 cells, the NCI-H1819-pBABE-SMARCA4-FLAG cell line had lower HURP levels (Fig. 5d). Furthermore, our panel of seven *SMARCA4* mutant NSCLCs exhibited higher levels of HURP compared with 19 *SMARCA4* wild-type NSCLC or HBEC lines, which had much lower levels of HURP protein (Fig. 5e,f).

As SMARCA4, as a component of the SWI/SNF complex, regulates gene transcription, we explored whether *HURP* transcription correlates with SMARCA4 expression. However, whole-transcriptome shotgun sequencing (RNA sequencing) and Illumina HumanWG-6 BeadChip microarray analyses revealed that our panel of NSCLC lines had no significant differences in mRNA levels (Supplementary Figs 4b and 5b, Supplementary Data 4, 5 and 7) or gene copy numbers of *HURP* (Supplementary Fig. 3a, Supplementary Data 3) although they had significantly increased levels of HURP protein (Fig. 5e,f). The same analyses were performed for *AURKA*, *TPX2* and *RAN*, but no correlation was obtained between SMARCA4 inactivation and gene copy numbers, transcription or protein levels of *AURKA*, *TPX2* and *RAN* in these cell lines (Supplementary Figs 3b–d, 4c–e, 5c–e and 6a–d, Supplementary Data 3–5). However, *SMARCA4* transcripts, serving as the positive controls, were decreased in NSCLCs harbouring mutations that inactivated SMARCA4 in comparison to the NSCLC lines expressing wild-type SMARCA4 (Supplementary Figs 3a and 4a). Additionally, we queried The Cancer Gene Atlas (TCGA) whole-genome and RNA sequencing databases. Lung adenocarcinomas harbouring *SMARCA4* mutations, including missense mutations, had lower levels of *SMARCA4* transcripts (*P*<0.01). In TCGA lung adenocarcinoma data sets there was a small, statistically significant increase in *HURP*, *AURKA*, *TPX2* or *RAN* mRNA levels correlating with SMARCA4 mutations of all types (Supplementary Fig. 7a,b). Having higher levels of HURP protein but comparable levels of its transcripts in *SMARCA4*-null NSCLC lines suggests that SMARCA4 might influence regulators of HURP protein translation or degradation, rather than regulating its transcription directly.

We also investigated whether VX-680 caused toxic effects by decreasing the protein levels of HURP. However, VX-680 treatment did not change HURP protein expression (Supplementary Fig. 8a). Taken together, our data suggest that SMARCA4 inactivation might reprogram mitotic spindle formation to dependency on centrosome-independent machinery that requires HURP activity. To investigate this, we stained centrosomes in NCI-H1819 cells with antibodies to PCM1, a centrosomal matrix marker. In immortalized HBEC-KT cells, 80% of the cells contain a single perinuclear spot brightly staining for PCM1, whereas in NCI-H1819 many cells lacked PCM1 staining and less than half the cells scored as having normal PCM1 staining (Supplementary Fig. 11).

## Discussion

NSCLCs, as well as several other cancer types, frequently carry homozygous *SMARCA4*-inactivating mutations^8^,^36^,^37^,^38^. The precise contributions of SMARCA4 loss to tumour initiation and/or progression are largely unknown. However, the observation that loss of SMARCA4 function affects 5% of the mammalian transcriptome^19^ suggests that it might create unique vulnerabilities in tumours that could be exploited for therapy. Knocking out *Smarca4* causes mitotic catastrophe in primary mouse fibroblasts and greatly decreases proliferation^24^. Obviously cancer cells avoid this through some compensatory mechanism. Here we show that human NSCLCs with *SMARCA4*-inactivating mutations are hypersensitive to inhibition of AURKA, a mitotic kinase required for bipolar spindle assembly. Loss of SMARCA4 is necessary for this hypersensitivity, but as re-expressing SMARCA4 in a cancer cell line lacking it only partially rescued cells from AURKA knockdown, SMARCA4 loss is not sufficient for this sensitivity. We speculate that the other alterations required for cancer cells to survive loss of SMARCA4 may also contribute to sensitivity to AURKA inhibitors.

The expression of Aurora A is often elevated in tumours and the *AURKA* gene maps to a locus (20q13) that is frequently amplified in some cancer types^39^,^40^,^41^,^42^. *SMARCA4* is mutated or deleted in more cancer types than NSCLC^36^,^37^,^43^, and a relationship between SNF5 mutations and AURKA was reported for rhabdoid tumours^44^. Thus vulnerability to AURKA inhibitors might not be limited to the loss of SMARCA4 in NSCLCs but might occur in other cancers with mutations in *SMARCA4* or other SWI/SNF components that cause a loss of SWI/SNF function.

In our primary screen, *AURKA* was not selected as a gene of interest because siRNA pool for *AURKA* did not meet our arbitrary criteria for biological significance (Supplementary Data 1). As we found that a rather complete knockdown of AURKA was required for cell toxicity (Fig. 3b), it is likely that the pooled siRNAs did not achieve this in the primary screen. Similarly, losses of SMARCA2 (refs 45, 46) and EZH2 (refs 47, 48) have been reported to be synthetically lethal with loss of SMARCA4. Both SMARCA2 and EZH2 siRNAs were toxic in our primary screen, but with *z*-scores that did not reach the −3 s.d. threshold we used for follow-up analysis (Supplementary Data 1). Knockdown of *SMARCA2* in a SMARCA4-null background inhibits cell growth over a period longer than the 96 h of our siRNA assay, probably explaining why a partial effect was seen. In Fig. 5, we observed that knocking down HURP decreased cell growth in NCI-H1819, but siRNA targeting HURP (DLGAP5) was not toxic in our primary screen (Supplementary Data 1). False-negative results are known to occur in genome-wide siRNA screens, and thus retesting all components of protein complexes identified in the primary screen by siRNAs to other components is warranted.

Additionally, it was previously reported that cancers driven by amplified *MYC* demonstrated sensitivity to AURKA inhibitors^49^,^50^,^51^. We checked the gene copy number, mRNA transcript and protein expression levels of MYC in our panel of NSCLC and HBEC lines and did not find a correlation between loss of *SMARCA4*, amplification of MYC and differential sensitivity to VX-680 (Supplementary Data 4–6, Supplementary Figs 9a–e and 7b).

To investigate whether the difference in sensitivity to AURKA depletion or to VX-680 is due to a general vulnerability to the inhibition of mitosis caused by differences in cell growth rates, we measured the cell doubling times of our panel of SMARCA4-null and SMARCA4-wild-type cell lines (Supplementary Table 1). The average doubling time for SMARCA4-null lines was 35±14 h and for SMARCA4 wild type was 44±8 h, (*P*=0.08 by Student's two-tailed *T*-test). However, the cell line that we chose as the representative of *SMARCA4* mutant NSCLCs, NCI-H1819, has a slow growth rate with an average doubling time of 53 h, which is higher than the mean doubling time of the NSCLC lines harbouring wild-type *SMARCA4*. We also used siRNAs against the mitotic kinase PLK1, and antimitotic drugs Paclitaxel and GSK923295 as controls, to detect possible cell doubling rate-related effects. However, our results showed that SMARCA4-inactivated NSCLCs were only hypersensitive to the depletion or inhibition of AURKA.

Mitosis has been an important target for anticancer therapy. Antimitotics consist of conventional chemotherapeutic agents that alter microtubule dynamics such as vinca alkaloids, taxanes or epothilones and novel classes of antineoplastic drugs targeting the regulatory system that controls mitosis, such as Aurora, Polo-like or Cyclin-dependent kinases or Kinespondin inhibitors^52^,^53^. Owing to the severe side effects of cytotoxic microtubule poisons in clinical use, therapies targeting the molecular regulators of mitotic spindle machinery offer an alternative and potentially safer way to treat patients.

**Figure 6:**
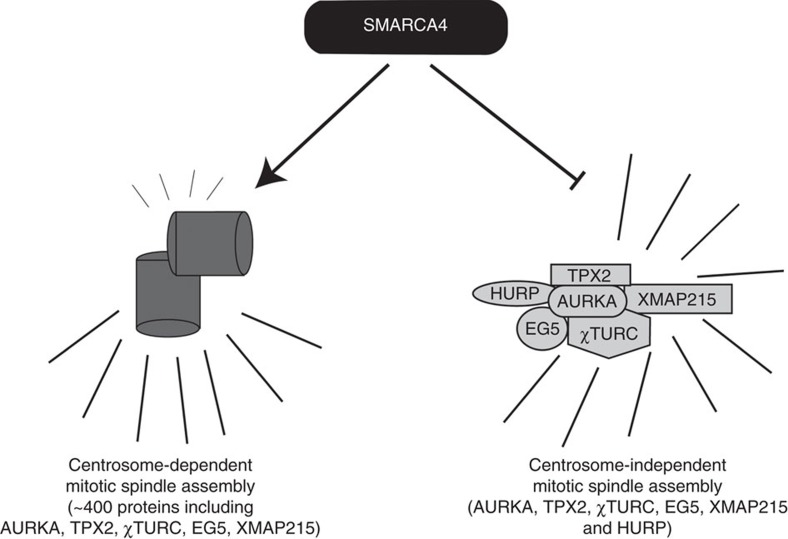
Proposed model for the relationship between SMARCA4 and mitotic spindle assembly machineries. SMARCA4 may be necessary for the expression of a key component of the centrosome-dependent pathway for mitotic spindle formation and repress the centrosome-independent pathway of spindle formation. When SMARCA4 is lost, lung cancer cells upregulate HURP and become sensitive to its depletion. As HURP is a unique component of the centrosome-independent pathway normally dispensible for somatic cells, we speculate that hypersensitivity to Aurora kinase inhibitors is linked to a switch in the mechanisms for mitotic spindle formation.

There are two known cellular mechanisms for assembling mitotic spindles (Fig. 6). The centrosome-dependent mitotic spindle machinery is responsible for proper chromosome distribution during mammalian somatic cell division^54^. However, in plants and vertebrate oocytes mitotic spindles can also be formed in a chromosome-dependent manner in the absence of centrosomes^55^,^56^. Recently, some of the components of chromosome-dependent mitotic spindle machinery were identified^19^. All but one, HURP, are shared with the centrosome-dependent system. HURP is a microtubule bundling protein that is necessary to arrange and align the microtubules properly from chromosomes to the poles of cells^33^. *Hurp* knockout mice, unlike mice deficient in other mitotic spindle regulators, show normal development with the exception that females are infertile^35^. Thus HURP is not required in centrosome-harbouring mammalian somatic cells. Additional research on the female mice lacking *Hurp* gene expression demonstrated that oocytes, which lack centrosomes naturally, were not capable of dividing properly^57^, proving that HURP is critical for centrosome-independent mitotic spindle formation. Here we report that HURP was necessary for the viability of NCI-H1819 cells and this requirement was significantly reduced when *SMARCA4* expression was restored. HURP expression was elevated in NSCLC cell lines lacking SMARCA4, whereas it was very low or absent in NSCLC lines expressing wild-type SMARCA4. Moreover, *SMARCA4*-deficient NCI-H1819 cells show dramatic centrosomal abnormalities, which mainly include their total loss from the cells (Supplementary Fig. 11a,b). Although it is not known how SMARCA4 regulates centrosomes, these data suggest that SMARCA4 loss impairs centrosome function in mitotic spindle assembly and makes cell division largely chromosome-dependent, creating a therapeutic opportunity linked to a biomarker.

In addition to *HURP*, *TPX2* and *AURKA*, we report a validated collection of genes that potentially regulate the survival and/or proliferation of NCI-H1819 cells. Although some of these have established links to drugs or inhibitors, others are less characterized but offer a platform for future detailed functional studies. This list of selectively toxic genes, when depleted, may lead to novel vulnerabilities related to the status of *SMARCA4* or to other genes altered in the NCI-H1819 cell line.

## Methods

### Cell lines

NSCLC and immortalized HBEC lines were generated by the laboratories of John Minna and Adi Gazdar and their identities have been confirmed by short tandem repeat analysis of cellular DNA (Promega Corp, Madison, WI, USA). All cell lines were determined to be mycoplasma free by testing with the e-Myco Plus Mycoplasma PCR Detection Kit (Bulldog Bio, 2523448). NSCLC lines were cultured in RPMI-1640 medium (GIBCO, 11875) supplemented with 10% fetal bovine serum (v/v) (Atlanta Biological, S11550). HBEC lines were grown in Keratinocyte-SFM medium (GIBCO, 17005) supplemented with EGF and keratinocyte extract (provided by the manufacturer). NCI-H1819, A549, NCI-H1299, NCI-H157, NCI-H23, NCI-H661 NCI-HCC15 and NCI-H1355 lines harbour *SMARCA4*-inactivating mutations. NCI-H1792, NCI-H1975, NCI-H3255, NCI-H358, NCI-H820, HCC4006, HCC827 Calu-1, Calu-3, NCI-H1648, NCI-H2073, NCI-H2882, NCI-H3122, HCC1171, HCC193 and HCC78 express wild-type SMARCA4. HBEC3-KT, HBEC30-KT and HBEC34-KT are HBECs immortalized with stable expression of CDK4 and hTERT^58^.

### Reagents

VX-680 (Chemietek, CT-VX680) GSK923295 (ChemSene, CS-0056) Paclitaxel, (LC Labs, P-9600), Pemetrexed (LC Labs, P-7177), Etoposide (LC Labs, E-4488), Erlotinib (LC Labs, E-4497), Crizotinib (LC Labs, C-7900), Bortezomib (Alfa Aesar, J60378) and Brefeldin A (LC Labs, B-8500) was purchased in powder form and stored at 20 mM stock concentrations in dimethyl sulfoxide (DMSO) at −20 °C. TPX2 (Biolegend, mouse mAb, 628001, 1:1,000 dilution), AURKA (Cell Signaling, rabbit mAb, 4718, 1:1,000 dilution), Phospho-Histone H3 (Cell Signaling, rabbit mAb, 33770, 1:1,000 dilution), cleaved PARP (Cell Signaling, rabbit mAb, 9541, 1:1,000 dilution), FLAG (Cell Signaling, rabbit mAb, 2368, 1:1,000 dilution), SMARCA4/BRG1 (EMD Millipore, rat mAb, MABE60, 1:1,000 dilution), DLG7/HURP (Bethyl Laboratory, A300-852A, 1:1,000 dilution), c-MYC (Santa Cruz Biotechnology, mouse mAb, sc-40, 1:1,000 dilution) and β-Actin (MP Biomedicals, mouse mAb, 69100, 1:10,000 dilution) antibodies were used for immunoblotting assays. PCM1 (Cell Signaling, rabbit pAb, 5213, 1:500 dilution), Tubulin (Abcam, rat mAb, ab6160, 1:500 dilution), Alexa Fluor 488 goat anti-rat (ThermoFisher, A-11006, 1:1,000 dilution) and Alexa Fluor 568 goat anti-rabbit (ThermoFisher, A-11011, 1:1,000 dilution) antibodies were used for immunofluorescence assay. pBABE-SMARCA4-FLAG plasmid was a gift from Robert Kingston (Addgene plasmid no. 1959). This plasmid was initially reported in Sif *et al*.^59^ and contains the full-length SMARCA4-coding region. pBABE-Empty plasmid was a gift from the laboratory of Jerry Shay (UT Southwestern Medical Center at Dallas, TX, USA).

### High-throughput genome-wide siRNA library screen

The siRNA library was purchased from Dharmacon (GE Healthcare, Inc.). The siRNA library is designed to have 21,124 pools of 4 individual siRNAs targeting approximately 85% of all identified genes in the human genome. Transfection conditions for the NCI-H1819 cell line were optimized with non-targeting siRNAs and siRNAs targeting PLK1 to achieve minimal nonspecific toxicity and maximum specific toxicity, respectively. For each gene target, siRNA pools were arrayed in 96-well plates in columns 2–7. Columns 1 and 8 contained transfection reagent only. A reverse transfection protocol was employed to maximize the transfection efficacy. The siRNA pools (final concentration of 50 nM) were mixed with RNAiMax transfection reagent (0.23 μl per well) (Life Technologies, 13778-150) in OptiMEM medium (GIBCO, 31985) according to the optimized conditions. A total of 3,000 cells were dispensed into wells with an automated liquid dispenser (BioTek MicroFlo; Thermo-Fisher, Inc.). Each pool was screened in triplicate. Cells were incubated for 96 h at 37 °C in the presence of 5% CO_2_. Afterward, the culture medium was removed by centrifuging the plates upside down at 30*g* for 1 min in a liquid-collecting container and cell viability was measured with CellTiter-Glo (Promega, G7573) assay using the manufacturer's protocol.

### Secondary low-throughput confirmation screen

A custom siRNA library with pools of 4 individual siRNAs targeting each of the 38 human genes was obtained from Dharmacon (GE Healthcare, Inc.). Transfection conditions and methods were the same as in the high-throughput screen. siRNAs of interest were defined on the basis of statistical and biological significance. Statistical significance was defined as a *P* value of <0.01 by one-way ANOVA and *post hoc* Dunnet's multiple comparison tests. Biological significance was arbitrarily defined as a 50% decrease in the cell viability after 5 days measured by a CellTiter-Glo assay, compared with the mean value obtained for cells transfected with non-targeting siRNA. Hits identified as statistically and biologically significant were investigated in the follow-up experiments.

### Microplate apoptosis assay

Transfections of the siRNA pools were performed in the same manner with the high- and low-throughput library screens in 96-well plate format. Seventy-two hours after transfections, culture medium was removed by centrifuging the assay plates upside down in liquid-collecting containers at 30*g* for 1 min. Caspase 3 and/or 7 activity was assessed with Caspase-Glo 3/7 assay purchased from Promega (G8090). siRNAs demonstrating a *P* value of <0.01 by a one-way ANOVA and *post hoc* Dunnet's multiple comparison tests were considered statistically significant when compared with non-targeting siRNA-transfected cells.

### siRNA confirmations with cell viability assays

Individual or pooled siRNAs used for knockdowns of TPX2, AURKA, HURP and PLK1 were purchased from Dharmacon. They were dissolved in DNAse/RNase-free water overnight to make a final concentration of 5 μM. Reverse transfection was used to maximize the transfection efficacy. siRNA pools at a final concentration of 50 nM were mixed with RNAiMax transfection reagent (Life Technologies, 13778-150) in OptiMEM medium and dispensed into wells into which was added 1,000–2,000 cells. After incubating the transfected cells for 120 h, the culture medium was removed by centrifuging the plates upside down at 30*g* for 1 min in a liquid-collecting container and cell viability was measured with CellTiter-Glo assay. Triple biological replicates were tested for each siRNA.

### Generating SMARCA4-expressing cell lines

pBABE-Empty or pBABE-SMARCA4-FLAG plasmids were transfected into Phoenix retroviral producer cells and the supernatants containing retroviruses were collected. NCI-H1819 cells were infected and those containing retroviruses selected with puromycin. The cells were expanded as heterogeneous populations. The expression of SMARCA4 was confirmed by western blotting with monoclonal FLAG and SMARCA4 antibodies.

### Western blotting

In all, 2 × 10^5^ cells were transfected with individual siRNAs at the concentration of 50 nM in six-well format. Seventy-two hours after transfection, the culture medium was aspirated and cells were washed with PBS. Cells were lysed in RIPA lysis buffer (50 mM Tris buffer pH 8, 150 mM NaCl, 1% NP-40, 0.5% sodium, 0.1% SDS, 0.5% Sodium deoxycholate) supplemented with protease and phosphatase inhibitors. The protein concentrations of cell lysates were measured with the Protein DC assay (Bio-Rad, 500-0111). Endogenous TPX2, AURKA or HURP levels, phosphorylation of histone H3 and β-Actin expression as internal control were monitored by immunoblotting with monoclonal antibodies.

NSCLC cells and HBECs, when 70–80% confluent, were lysed in RIPA lysis buffer (50 mM Tris buffer pH 8, 150 mM NaCl, 1% NP-40, 0.5% sodium, 0.1% SDS, 0.5% Sodium deoxycholate) supplemented with protease and phosphatase inhibitors. The protein concentrations of cell lysates were measured with the Protein DC assay. Endogenous SMARCA4, HURP, AURKA, TPX2, RAN or MYC levels and β-Actin expression as internal control were monitored by immunoblotting with monoclonal antibodies. Protein band intensities were measured with the ImageQuant software.

Uncropped scans of all blots are provided in Supplementary Fig. 12.

### Immunofluorescence assay

Cells on cover slips were fixed with 4% formaldehyde in PBS for 15 min at room temperature. After washing with PBS, cells were permeabilized with 0.25% Triton-X in PBS for 15 min at room temperature. After washing with PBS, cover slips were incubated with 10% BSA blocking solution for 1 h. Next cells were co-immunostained with anti-PCM1 and anti-Tubulin antibodies for 24 h. Samples were washed and further treated with Alexa Fluor secondary antibodies for 1 h. After washing, cover slips were mounted on microscope slides with Fluoroshield with DAPI (Sigma, F6057) mounting reagent. Microscopic observations were performed on an EVOS FL Cell Imaging System. Fractions of cells with centrosomal PCM1 within the total cell population (DAPI and Tubulin-positive cells) were calculated and graphed.

### Microplate drug sensitivity assays

NCI-H1819 cells (3,000 per well) were seeded in 96-well plates and incubated at 37 °C for 24 h. Treatment with vehicle (DMSO) or serial dilutions of the indicated compounds in 9 concentrations from 1 to 33,300 nM were performed in triplicate (final DMSO concentration=0.34% in all wells). After 120 h, media were removed and cell viability was measured with a CellTiter-Glo assay.

A dose–response study of a panel of NSCLC and HBEC cell lines with VX-680, Paclitaxel, GSK923295, Pemetrexed, Etoposide, Erlotinib, Crizotinib, Bortezomib and Brefeldin A was performed in 384-well plate format. Cells were plated at a density of 1,000–1,500 cells per well and incubated overnight at 37 °C as described above. Drugs/chemicals were dosed in DMSO at 12 concentrations ranging from 50 pM to 50 μM (half-log dilutions) in triplicate. After incubating the cells at 37 °C for 96 h, cell viability was measured with a CellTiter-Glo assay. The comparative tests of nine agents were performed twice on different days. Dose responses of each cell line to each agent were assessed and the EC50 values were calculated with the five-parameter logistic equation.

### Mouse xenograft studies

Cells derived from the human NSCLC lines NCI-H1299 and HCC827 were trypsinized and collected in RPMI 1640 media supplemented with 10% fetal bovine serum (v/v). The cells were centrifuged at 1,000*g* and the media was aspirated. After washing twice with sterilized PBS, cells were resuspended and diluted in PBS to have 2.5 × 10^6^ cells per 100 μl concentration. Single-cell suspensions (100 μl) were injected into the right flanks of 8–10-week-old, female NOD/SCID mice. Mice xenografted with NCI-H1299 and HCC827 cells were kept in a sterile vivarium maintained by the Animal Resource Center at UT Southwestern. Tumour volumes were measured twice a week with caliper measurements and calculated by the formula: tumour volume=(length × width^2^)/2. VX-680 treatment was initiated when tumours reached a mean volume of 150–200 mm^3^. Mice were assigned to treatment groups in serpentine style after sorting according to the tumour volume. No blinding was used in evaluating the experiment. VX-680 was formulated with 50% PEG-300 (Sigma, 90878) in PBS. For each dose of VX-680 four mice xenographed with each cell line were used. Mice were injected intraperitoneally with 0, 5, 10, 20, 30 and 50 mg kg^−1^ VX-680 twice daily. This number was chosen to limit the number of animals used and because differences in the responses of the two cell lines that were not statistically significant with this group size were felt to be biologically insignificant as well. The experiments were terminated when the largest tumours reached 1500, mm^3^. Animal studies were carried out under a protocol approved by the Institutional Animal Care and Use Committee of UT Southwestern that conforms to the requirements of Animal Act PL99-158 (as amended) and guidelines stated in the ‘Guide for the Care and Use of Laboratory Animals'.

### Gene copy number variation analysis

Genomic DNA or total RNA was extracted from cell lines by using the DNeasy Tissue Kit or RNeasy Plus Mini Kit (QIAGEN, Valencia, CA, USA). The cDNA was prepared by reverse transcription of RNA using the High-Capacity cDNA Reverse Transcription Kits (Applied Biosystems) according to the manufacturer's protocol.

### Whole-transcriptome shotgun sequencing (RNA sequencing)

The procedure for sequencing RNA and assessing quality control is described in detail in Wang *et al*.^60^

### RNA-Seq alignment and quantification

FastQC (Babraham Bioinformatics Institute) was used to check the sequencing quality, and high-quality reads were mapped to human reference genome (hg19) along with the gene annotation data (genecode v19) from the Genecode database using STAR (v2.4.2)^61^. RSeQC was applied for assessing RNA sample quality^62^. Gene-level expression was reported in fragments per kilobase per million reads by Cufflinks^63^. Raw RNA-seq data will be deposited to SRA database after the acceptance of this manuscript.

### Illumina humanWG BeadChip microarray analysis

A description of the microarray analysis is published^64^ and the data are deposited in GEO as GSE32036.

### TCGA tumour data sets

Genome and RNA sequencing data for lung adenocarcinoma were obtained from cBioportal database and search platform. The RSEM expression data was log2 transformed. *P* values provided by the database were used.

### Data availability

Primary data for this work are contained in Supplementary Data or at GEO as GSE32036.

## Additional information

**How to cite this article:** Tagal, V. *et al*. *SMARCA4*-inactivating mutations increase sensitivity to Aurora kinase A inhibitor VX-680 in non-small cell lung cancers. *Nat. Commun.* **8,** 14098 doi: 10.1038/ncomms14098 (2017).

**Publisher's note:** Springer Nature remains neutral with regard to jurisdictional claims in published maps and institutional affiliations.

## Supplementary Material

Supplementary Information Supplementary Figures and Supplementary Table

Supplementary Data 1 A table of cell viability results for high-throughput siRNA library screen of NCI-H1819 cells. This table includes gene ID, normalized viability to the median value of the corresponding microplate, mean value of all triplicates, z scores and robust z scores.

Supplementary Data 2 A table of cell viability results for high-throughput siRNA library screen of HBEC30-KT cells. This table includes genes, whose siRNA pools caused 25% or more cytotoxic effect on cell viability.

Supplementary Data 3 A table of drug sensitivity data and SMARCA4 mutation status of cell lines. This table includes cell line name, COSMIC ID for SMARCA4 mutation status (if exists), tissue type, cancer type and drug sensitivity data (mean of EC50 values).

Supplementary Data 4 A heatmap of gene copy number data of cell lines for SMARCA4, AURKA, HURP, TPX2, RAN and MYC. Red color for amplifications, green color for deletions and black color for normal gene copy numbers were used.

Supplementary Data 5 A heatmap of RNA content (RNA sequencing) of cell lines for SMARCA4, AURKA, HURP, TPX2, RAN and MYC. Signals are represented as log2(signal). Green (low) to red (high) color scale was used.

Supplementary Data 6 A heatmap of RNA content (Illumina HumanWG BeadChip microarray analysis) of cell lines for SMARCA4, AURKA, HURP, TPX2, RAN and MYC. Signals are represented as log2(signal). Green (low) to red (high) color scale was used.

Supplementary Data 7 A heatmap of RNA content (RNA sequencing) of cell lines for the whole genome. Signals are represented as log2(signal). White (low) to black (high) color scale was used.

## Figures and Tables

**Table 1:** Response of NCI-H1819 to AURKA inhibitors.

**Drug**	**EC50 (μM)**
VX-680	0.045
MLN8237	0.041
PHA-739358	0.105
ENMD-2076	0.569
VX-689	0.694

Dose response experiments were conducted with five AURKA inhibitors on NCI-H1819 cells and the EC50 values are shown.
