# Supplementary material for: SMARCA4-inactivating mutations increase sensitivity to Aurora kinase A inhibitor VX-680 in non-small cell lung cancers
Source: Nat Commun. 2017 Jan 19;8:14098. doi: 10.1038/ncomms14098 (PMC5253647; doi:10.1038/ncomms14098)
Supplement: Supplementary Information — Supplementary Figures and Supplementary Table [file ncomms14098-s1.pdf]

a

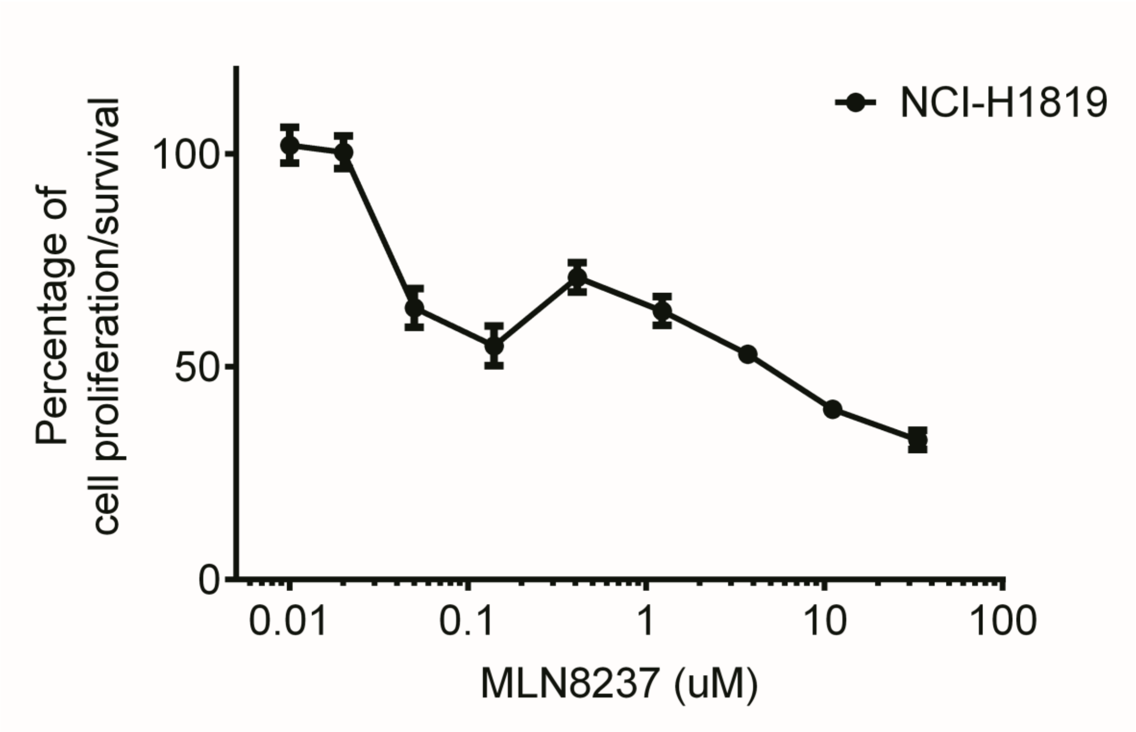

Supplementary Figure 1. MLN8237 treatments show an unusual camel-back response pattern. NCI-H1819 cells were treated with serial concentrations of MLN8237 and four days after treatment, cell viability was measured with a CellTiter-Glo assay. Each symbol indicates means and SD of sample groups.

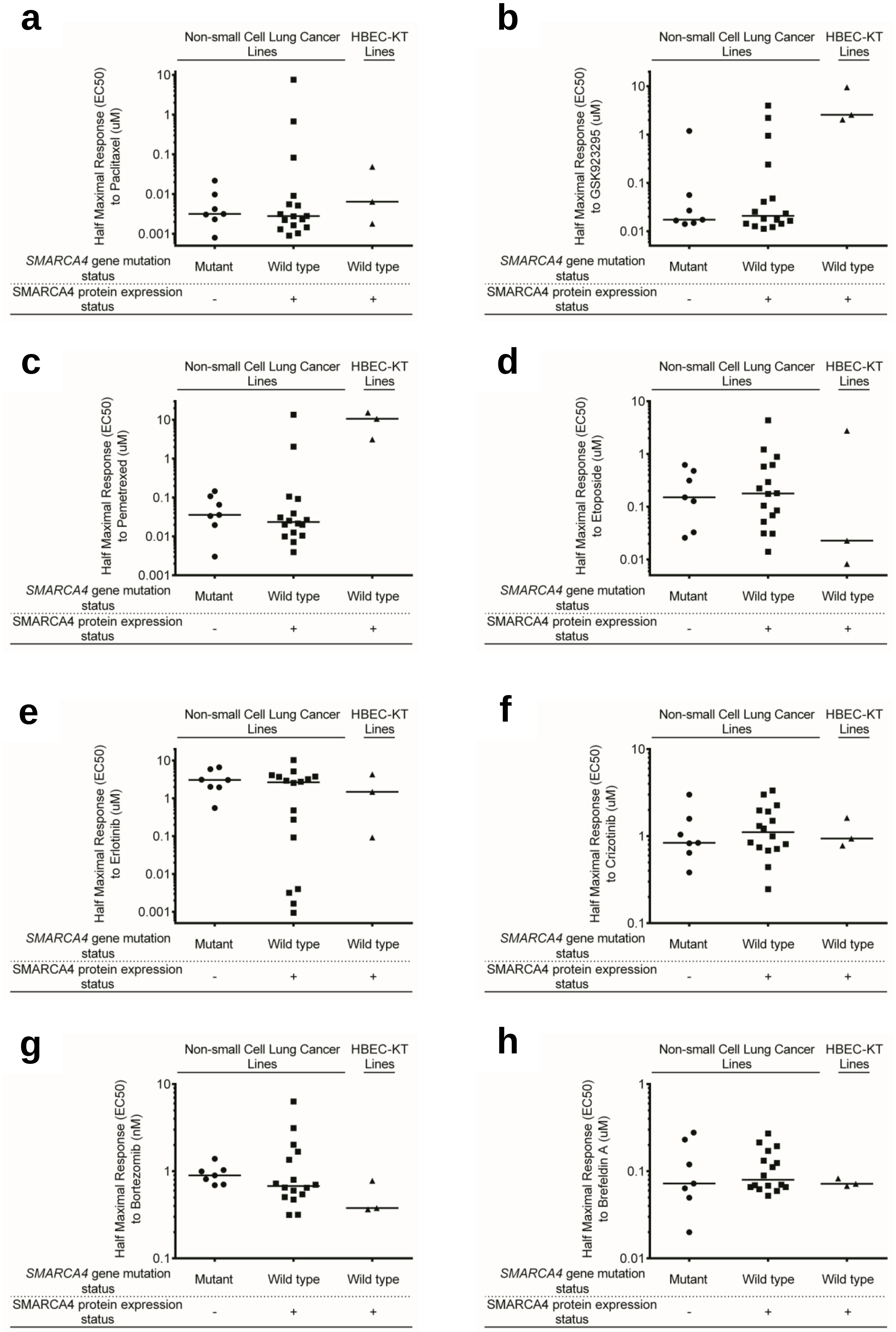
a
Non-small Cell Lung Cancer Lines
HBEC-KT Lines
Half Maximal Response (EC50) to Paclitaxel (uM)
10
1
0.1
0.01
0.001
SMARCA4 gene mutation status
Mutant
Wild type
Wild type
SMARCA4 protein expression status
-
+
+
b
Non-small Cell Lung Cancer Lines
HBEC-KT Lines
Half Maximal Response (EC50) to GSK923295 (uM)
10
1
0.1
0.01
SMARCA4 gene mutation status
Mutant
Wild type
Wild type
SMARCA4 protein expression status
-
+
+
c
Non-small Cell Lung Cancer Lines
HBEC-KT Lines
Half Maximal Response (EC50) to Pemetrexed (uM)
10
1
0.1
0.01
0.001
SMARCA4 gene mutation status
Mutant
Wild type
Wild type
SMARCA4 protein expression status
-
+
+
d
Non-small Cell Lung Cancer Lines
HBEC-KT Lines
Half Maximal Response (EC50) to Etoposide (uM)
10
1
0.1
0.01
SMARCA4 gene mutation status
Mutant
Wild type
Wild type
SMARCA4 protein expression status
-
+
+
e
Non-small Cell Lung Cancer Lines
HBEC-KT Lines
Half Maximal Response (EC50) to Erlotinib (uM)
10
1
0.1
0.01
0.001
SMARCA4 gene mutation status
Mutant
Wild type
Wild type
SMARCA4 protein expression status
-
+
+
f
Non-small Cell Lung Cancer Lines
HBEC-KT Lines
Half Maximal Response (EC50) to Crizotinib (uM)
10
1
0.1
SMARCA4 gene mutation status
Mutant
Wild type
Wild type
SMARCA4 protein expression status
-
+
+
g
Non-small Cell Lung Cancer Lines
HBEC-KT Lines
Half Maximal Response (EC50) to Bortezomib (nM)
10
1
0.1
SMARCA4 gene mutation status
Mutant
Wild type
Wild type
SMARCA4 protein expression status
-
+
+
h
Non-small Cell Lung Cancer Lines
HBEC-KT Lines
Half Maximal Response (EC50) to Brefeldin A (uM)
1
0.1
0.01
SMARCA4 gene mutation status
Mutant
Wild type
Wild type
SMARCA4 protein expression status
-
+
+

Supplementary Figure 2. Survey of a panel of NSCLC and HBEC lines reveals no hyper-sensitivity of SMARCA4-inactivated NSCLCs to various classes of anti-cancer agents and general toxic chemicals. A panel of NSCLC and HBEC lines was treated with **(a)** Paclitaxel, **(b)** GSK923295, **(c)** Pemetrexed, **(d)** Etoposide, **(e)** Erlotinib, **(f)** Crizotinib, **(g)** Bortezomib and **(h)** Brefeldin A for four days and cell viability was measured with a CellTiter-Glo assay. These experiments were performed twice and the mean EC50s of their individual responses are shown. Horizontal bars indicate medians of sample groups.

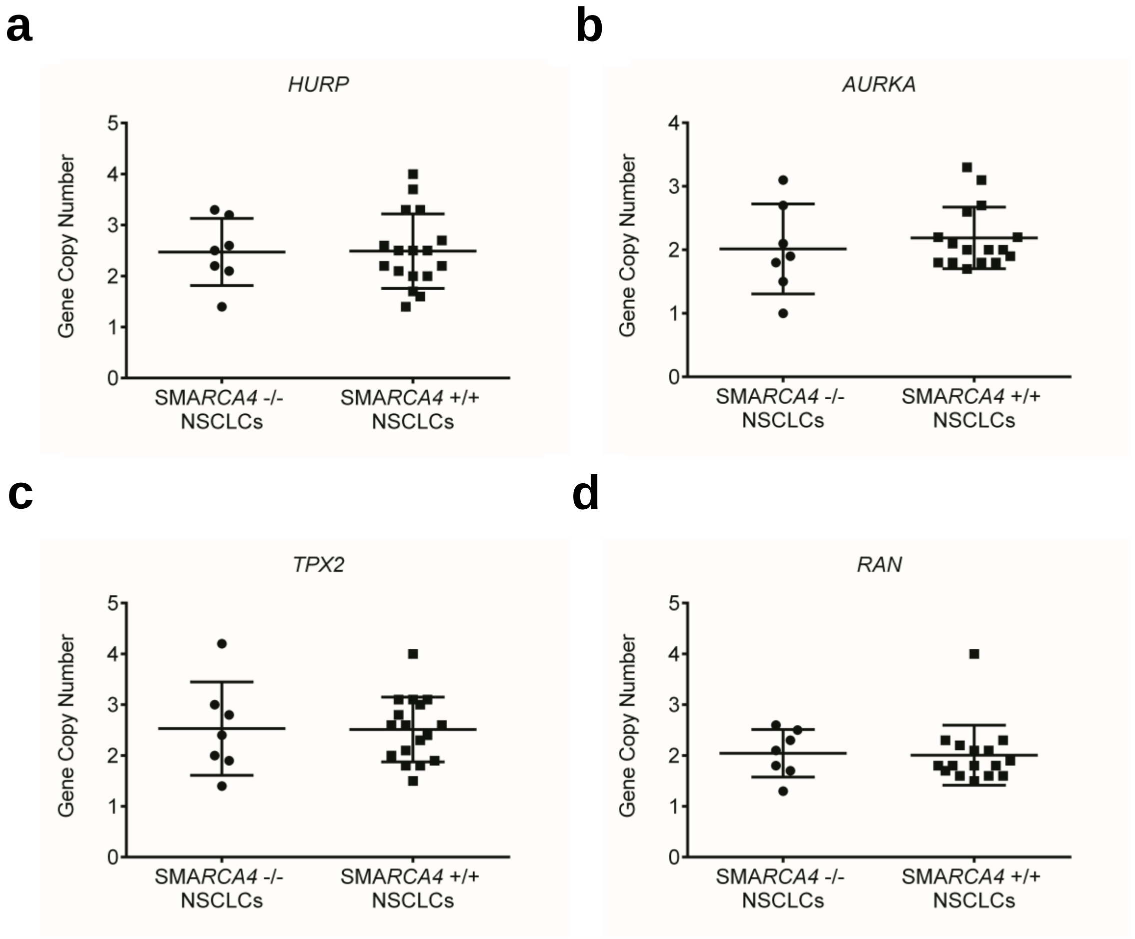

Supplementary Figure 3. Gene copy numbers of *HURP*, *AURKA*, *TPX2* and *RAN* do not vary among tested SMARCA4-null and wild-type NSCLCs. Gene copy numbers of **(a)** *HURP*, **(b)** *AURKA*, **(c)** *TPX2* and **(d)** *RAN* were measured. Each symbol represents the copy number for a cell line. Horizontal bars indicate means of sample groups with standard deviations.

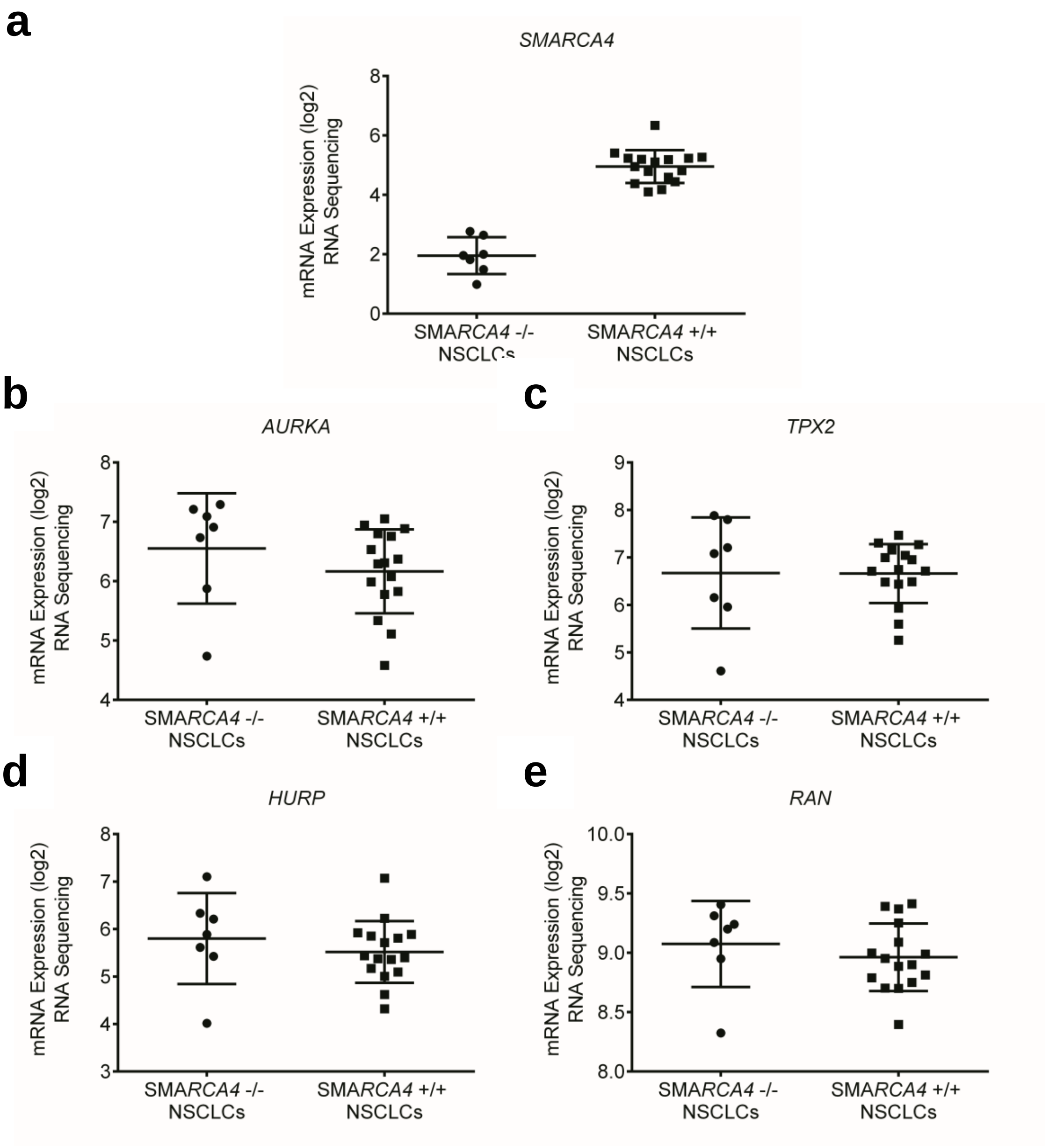

Supplementary Figure 4. SMARCA4 does not regulate the transcription of *HURP, AURKA, TPX2* or *RAN*. mRNA levels of **(a)** *SMARCA4*, **(b)** *HURP*, **(c)** *AURKA*, **(d)** *TPX2* and **(e)** *RAN* were assessed with RNA sequencing and individual transcript levels for each cell line are presented. Horizontal bars indicate means of sample groups with standard deviations.

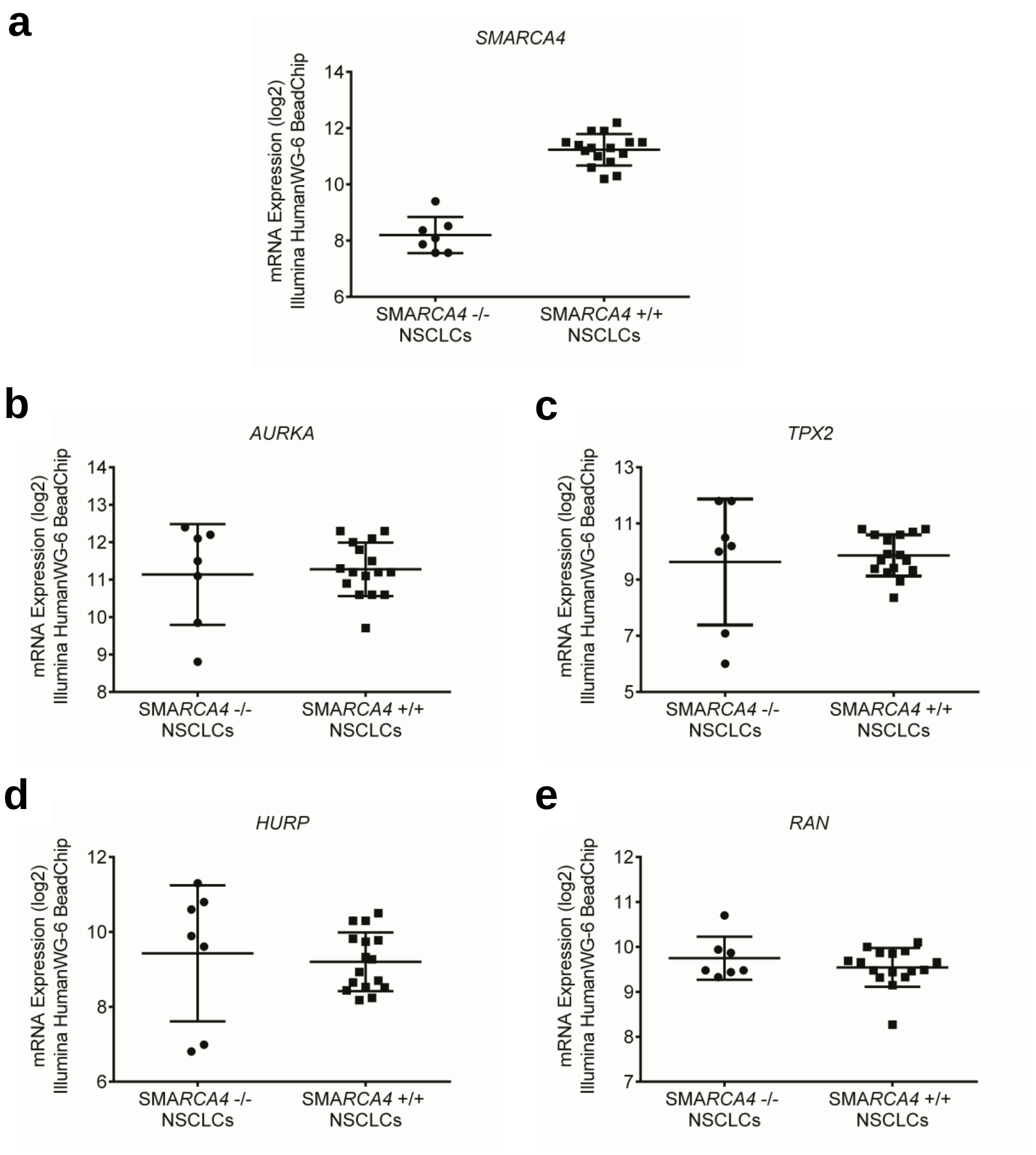

Supplementary Figure 5. SMARCA4 does not regulate the transcription of *HURP, AURKA, TPX2* or *RAN*. mRNA levels of **(a)** *SMARCA4*, **(b)** *HURP*, **(c)** *AURKA*, **(d)** *TPX2* and **(e)** *RAN* were assessed with Illumina HumanWG BeadChip microarray analysis and individual transcript levels for each cell line are presented. Horizontal bars indicate means of sample groups with standard deviations.

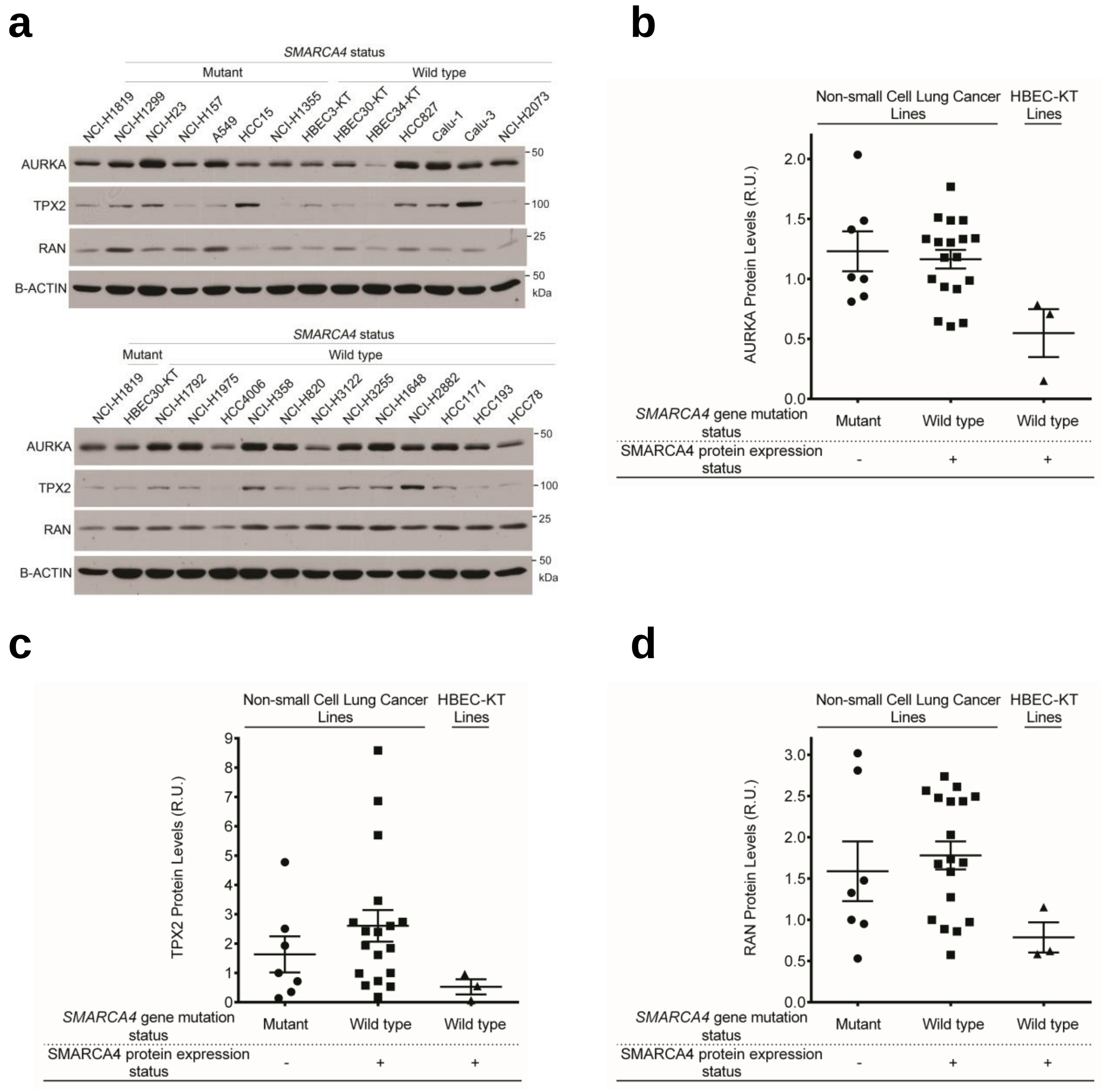

Supplementary Figure 6. SMARCA4 does not regulate the protein levels of AURKA, TPX2 or RAN. **(a)** AURKA, TPX2 and RAN protein expressions were measured in a panel of *SMARCA4*-mutant or wild type NSCLCs and HBECs by immunoblotting. **(b)** AURKA **(c)** TPX2, and **(d)** RAN protein band intensities were measured with ImageQuant software and graphed. Horizontal bars indicate medians of sample groups with standard deviations.

a

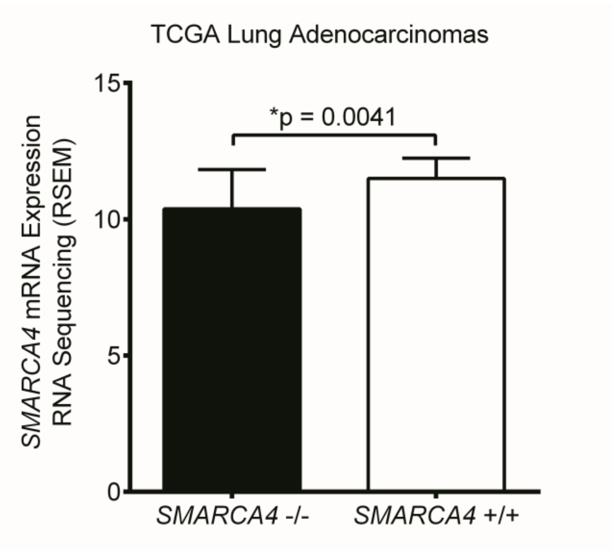

b

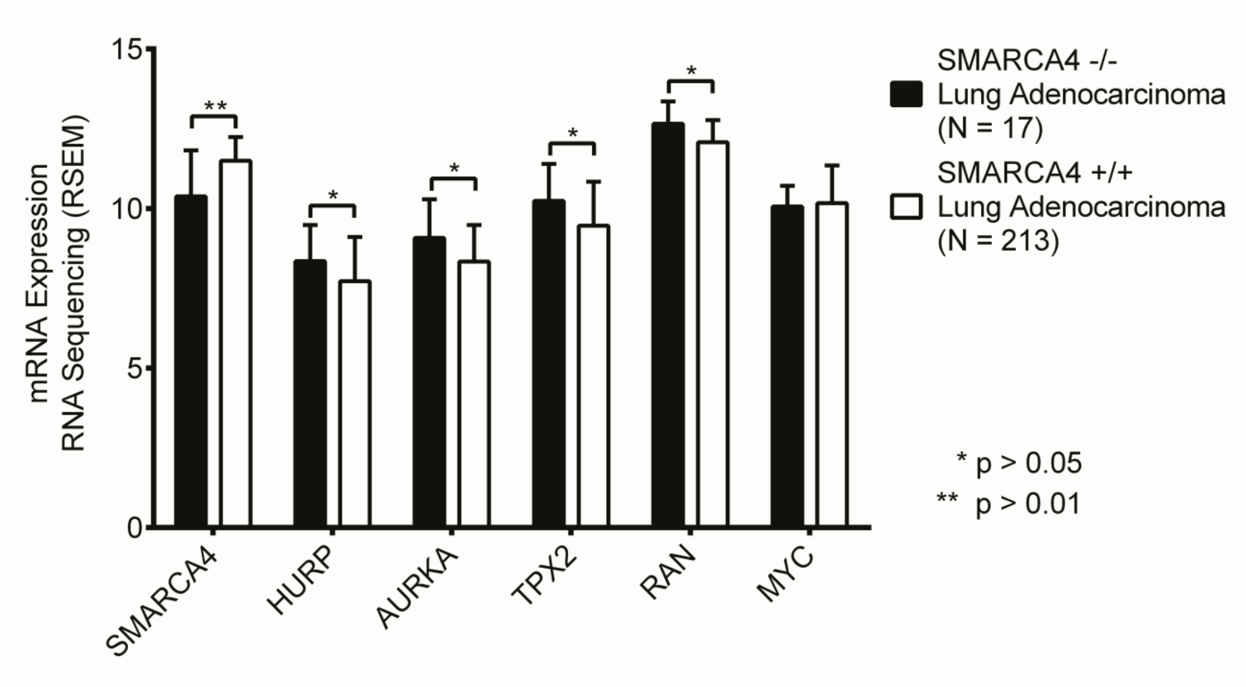

Supplementary Figure 7. SMARCA4 does not regulate the transcription of *HURP, AURKA, TPX2* or *RAN* in TCGA lung adenocarcinoma datasets. **(a)** Mean transcript levels of *SMARCA4* in *SMARCA4*-mutant or wild-type tumors from TCGA lung adenocarcinomas are graphed. Transcription data and their p-values were obtained from cBioPortal database. **(b)** mRNA levels of *HURP, AURKA, TPX2, RAN* and *MYC* were queried in TCGA lung adenocarcinoma datasets. Transcription data and their p-values were obtained from cBioPortal database.

a

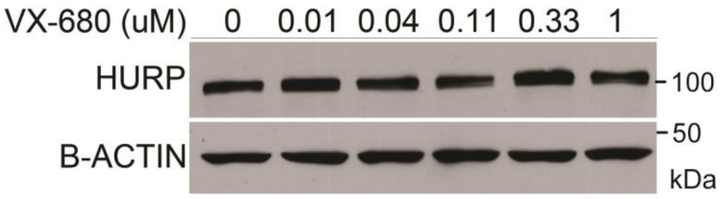

Supplementary Figure 8. VX-680 treatments do not have an effect on HURP protein levels. NCI-H1819 cells were treated with VX-680 and after 48 hours, cell lysates were collected. HURP protein levels were monitored by immunoblotting.

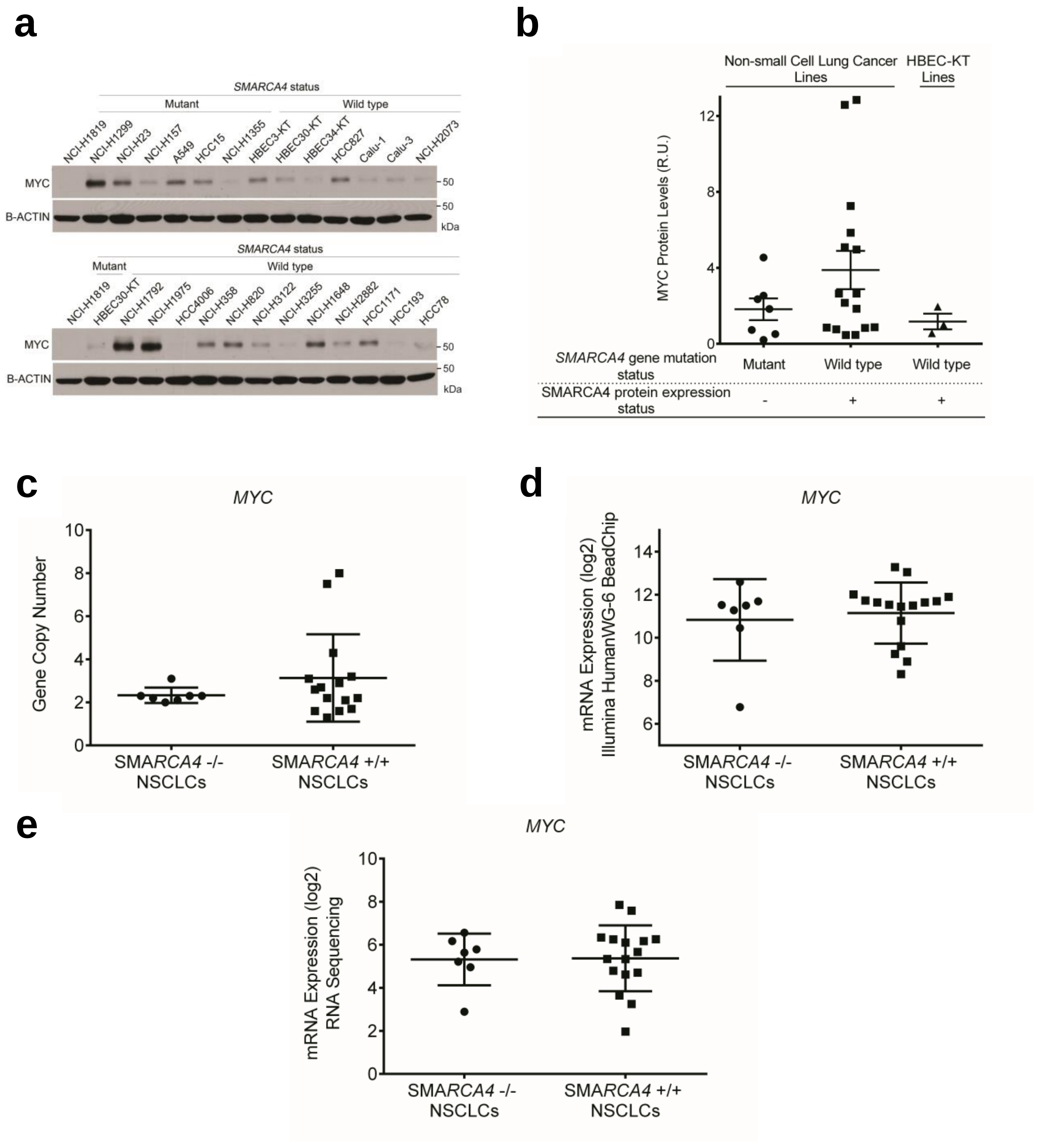

Supplementary Figure 9. MYC expression levels does not correlate with the mutation status of *SMARCA4* in NSCLCs. **(a)** MYC protein levels were monitored in a panel of *SMARCA4*-mutant or wild type NSCLCs and HBECs by immunoblotting. **(b)** MYC protein band intensities were measured with ImageQuant software and graphed. Horizontal bars indicate means of sample groups with standard errors. *MYC* was assessed for **(c)** gene copy numbers, **(d)** mRNA levels with RNA sequencing, **(e)** mRNA levels with Illumina HumanWG Beadchip microarray analysis, and individual levels for each cell line are presented. Horizontal bars indicate means of sample groups with standard deviations.

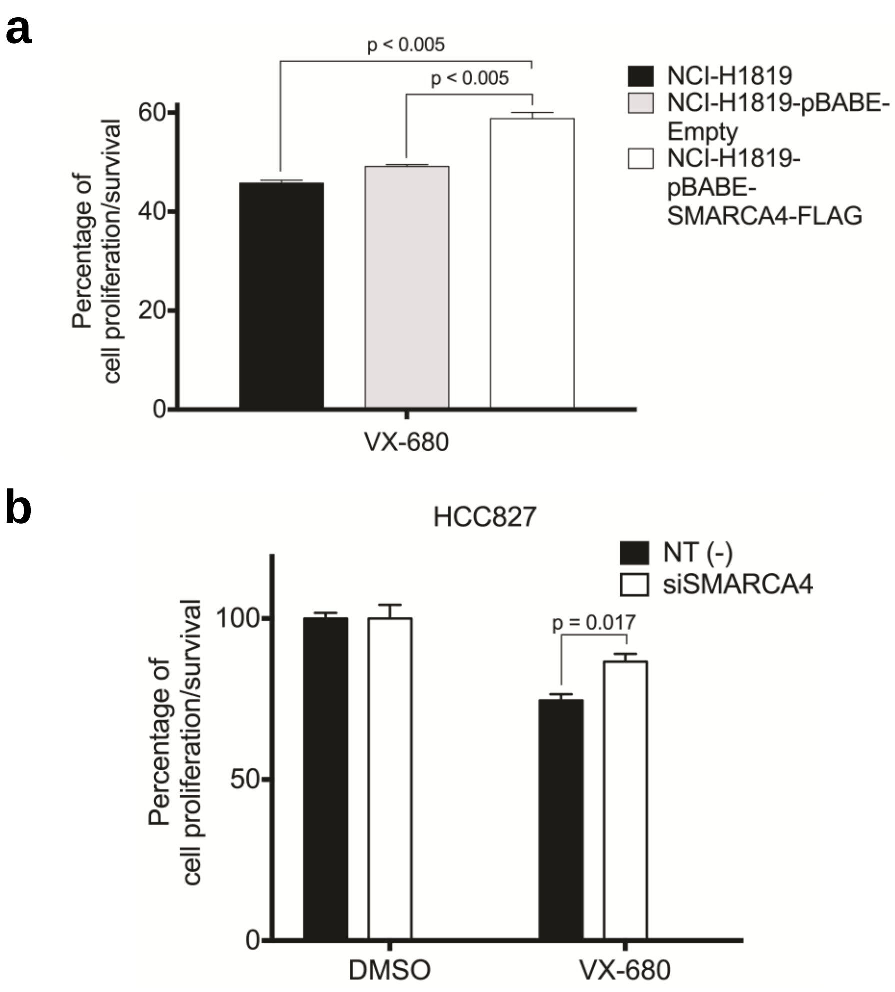

Supplementary Figure 10. Re-introduction of wild-type *SMARCA4* desensitizes against VX-680 toxicity whereas depletion of *SMARCA4* does not cause sensitivity to VX-680. **(a)** Five days after treatment with DMSO or 300 nM of VX-680 in NCI-H1819, NCI-H1819-pBABE and NCI-H1819-pBABE-SMARCA4-FLAG cells, cell viability was measured with a CellTiter-Glo assay on triplicate biological replicates. Statistical significance was assessed by a one-way ANOVA and post-hoc Dunnet's multiple comparison test. **(b)** Two days after transfecting with siRNA pools against *SMARCA4*, HCC827 cells were treated with DMSO or 300 nM of VX-680 for four days. Cell viability was measured with a CellTiter-Glo assay on triplicate biological replicates. Statistical significance was assessed by a two-sided unpaired T-test. Horizontal bars indicate means of sample groups with standard deviations.

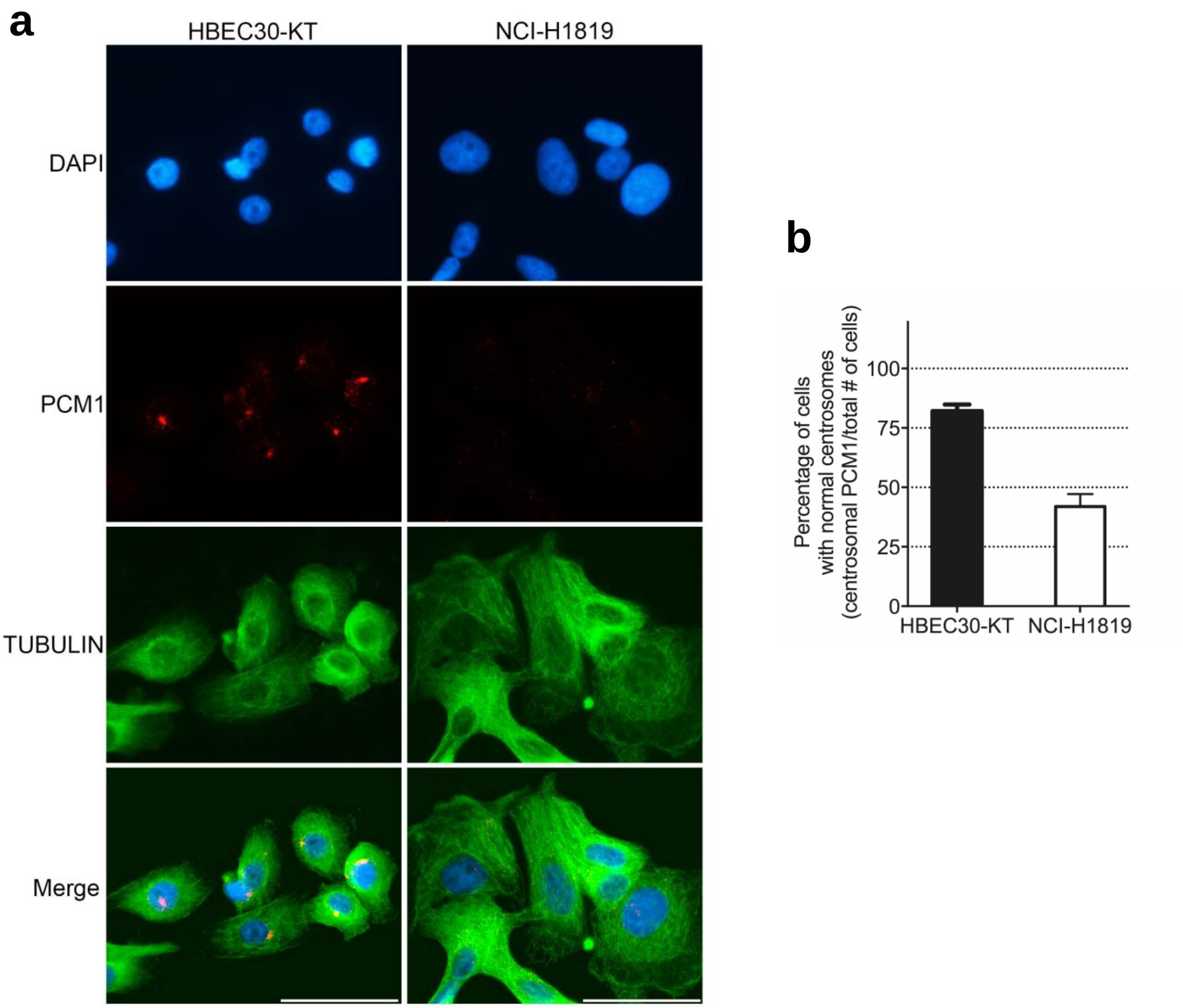

Supplementary Figure 11. *SMARCA4*-mutant NCI-H1819 cells show centrosomal defects. **(a)** HBEC30-KT and NCI-H1819 were immunostained against PCM1 as a centrosomal marker. DAPI and Tubulin were used to visualize the nuclei and cytoplasm, respectively. Scale bar, 100 um. **(b)** Fractions **of** cells with normal centrosomal PCM1 were calculated and graphed from two independent experiments (n=103 for HBEC30-KT, n=102 for NCI-H1819). Horizontal bars indicate the mean of individual images with SEM.

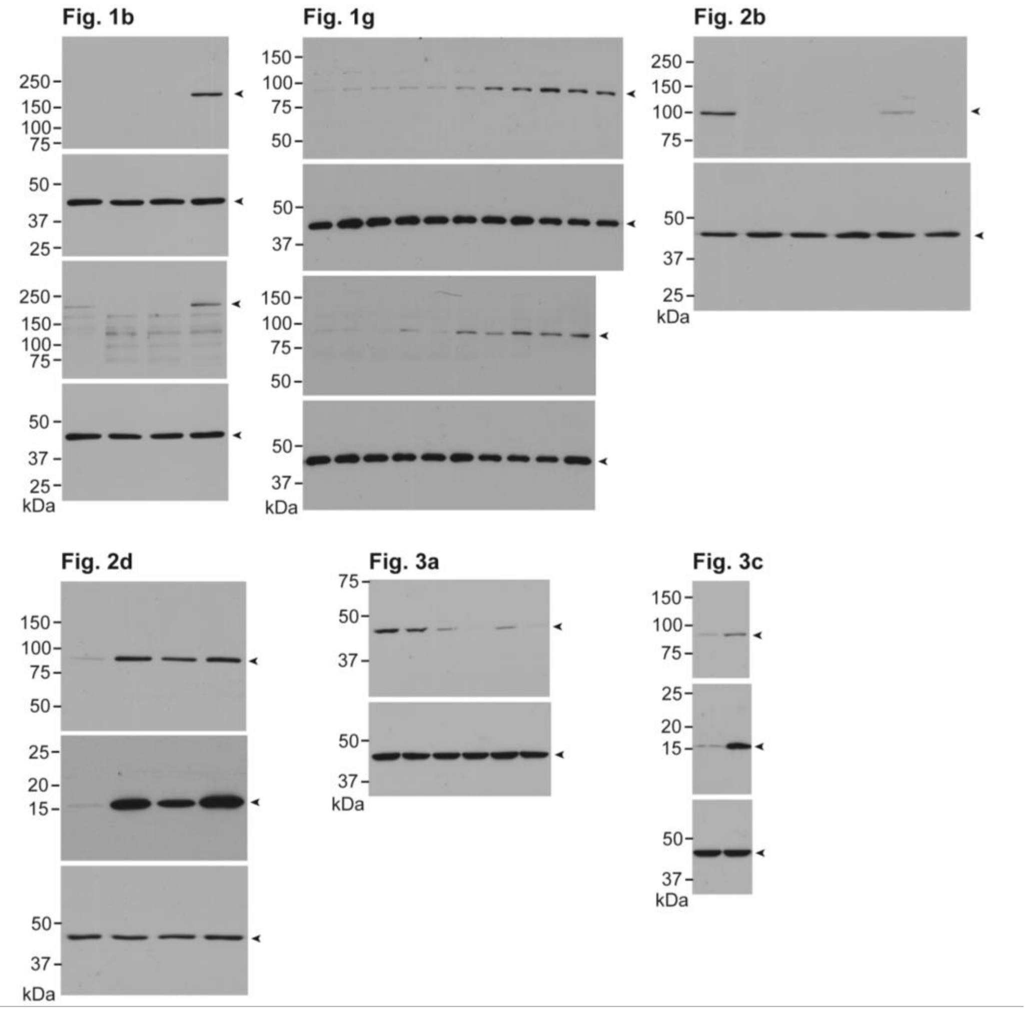

Supplementary Figure 12. Full gel images for figures 1 to 3.

Fig. 4b

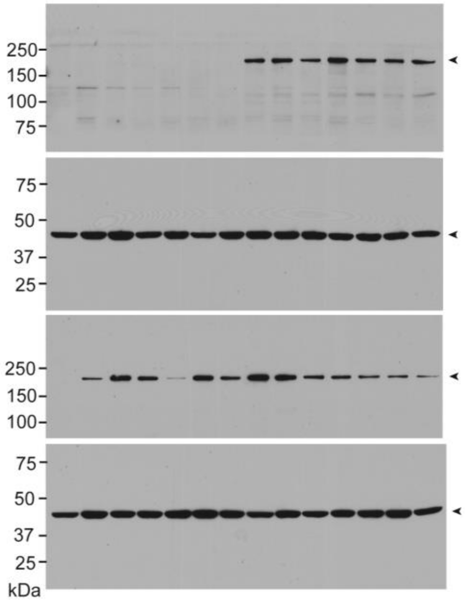

Fig. 5a

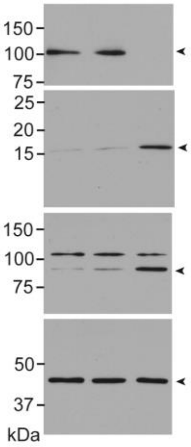

Fig. 5d

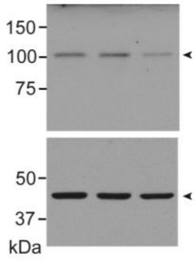

Fig. 5e

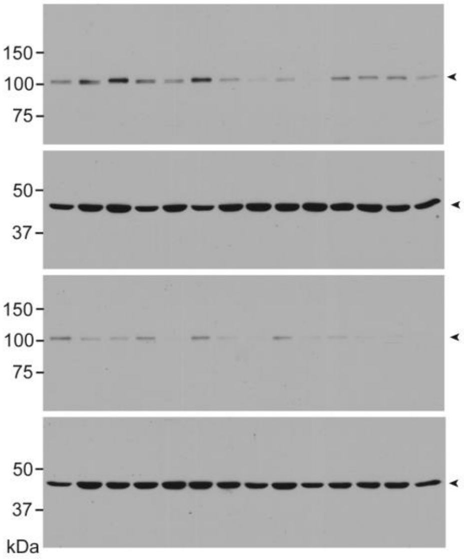

Supplementary Figure 12 (cont'd). Full gel images for figures 4 to 5.

Fig. S6a

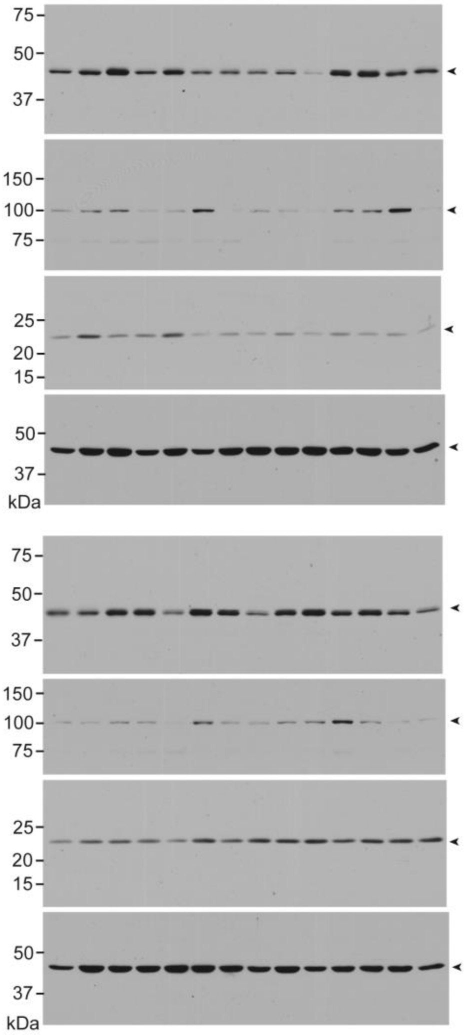

Fig. S8a

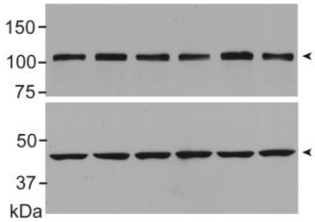

Fig. S9a

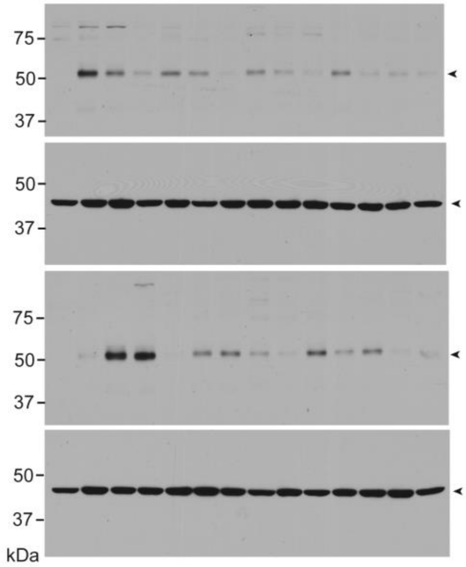

Supplementary Figure 12 (cont'd). Full gel images for supplementary figures 1 to 10.

Supplementary Table 1. Mean EC50s of the response to VX-680 and doubling times for NSCLC cell lines grouped by presence or absence of SMARCA4 (*Columns in white: SMARCA4-mutant, protein expression validated (-) NSCLC lines, Columns in grey: SMARCA4 wild-type, protein expression validated (+) NSCLC lines*)

***SMARCA4*-mutant**			***SMARCA4* wild-type**		
Cell line	Doubling time (hr)	EC50 (uM)	Cell line	Doubling time (hr)	EC50 (uM)
NCI-H1819	51.3	0.057	HCC827	44.5	2.134
NCI-H1299	22.5	0.332	Calu-1	49.8	1.024
NCI-H23	39.1	0.263	Calu-3	40.1	0.548
NCI-H157	20.8	0.056	NCI-H2073	45.3	2.938
A549	23.9	0.080	NCI-H1792	32.6	0.774
HCC15	29.1	0.057	NCI-H1975	42.2	0.600
NCI-H1355	48.8	0.030	HCC4006	46.8	4.336
			NCI-H358	38.0	0.552
			NCI-H820	65.2	3.078
			NCI-H3122	48.5	21.050
			NCI-H3255	NA	0.053
			NCI-H1648	41.9	1.769
			NCI-H2882	34.8	1.011
			HCC1171	47.5	1.290
			HCC193	45.5	2.493
			HCC78	38.7	1.987
